# In situ correlation between metastable phase-transformation mechanism and kinetics in a metallic glass

**DOI:** 10.1038/s41467-021-23028-9

**Published:** 2021-05-14

**Authors:** Jiri Orava, Shanoob Balachandran, Xiaoliang Han, Olga Shuleshova, Ebrahim Nurouzi, Ivan Soldatov, Steffen Oswald, Olof Gutowski, Oleh Ivashko, Ann-Christin Dippel, Martin v. Zimmermann, Yurii P. Ivanov, A. Lindsay Greer, Dierk Raabe, Michael Herbig, Ivan Kaban

**Affiliations:** 1 IFW Dresden, Institute for Complex Materials, Dresden, Germany; 2 Max-Planck-Institut für Eisenforschung, Düsseldorf, Germany; 3 IFW Dresden, Institute for Metallic Materials, Dresden, Germany; 4 Institute of Natural Sciences, Ural Federal University, Ekaterinburg, Russia; 5 Deutsches Elektronen-Synchrotron DESY, Hamburg, Germany; 6 Department of Materials Science and Metallurgy, University of Cambridge, Cambridge, UK; 7 Present Address: Faculty of Environment, Jan Evangelista Purkyne University in Usti nad Labem, Usti nad Labem, Czech Republic

**Keywords:** Phase transitions and critical phenomena, Glasses, Characterization and analytical techniques

## Abstract

A combination of complementary high-energy X-ray diffraction, containerless solidification during electromagnetic levitation and transmission electron microscopy is used to map in situ the phase evolution in a prototype Cu-Zr-Al glass during flash-annealing imposed at a rate ranging from 10^2^ to 10^3^ K s^−1^ and during cooling from the liquid state. Such a combination of experimental techniques provides hitherto inaccessible insight into the phase-transformation mechanism and its kinetics with high temporal resolution over the entire temperature range of the existence of the supercooled liquid. On flash-annealing, most of the formed phases represent transient (metastable) states – they crystallographically conform to their equilibrium phases but the compositions, revealed by atom probe tomography, are different. It is only the B2 CuZr phase which is represented by its equilibrium composition, and its growth is facilitated by a kinetic mechanism of Al partitioning; Al-rich precipitates of less than 10 nm in a diameter are revealed. In this work, the kinetic and chemical conditions of the high propensity of the glass for the B2 phase formation are formulated, and the multi-technique approach can be applied to map phase transformations in other metallic-glass-forming systems.

## Abstract (web summary)

The competition between the formation of different phases and their kinetics need to be clearly understood to make materials with on-demand and multifaceted properties. Here, the authors reveal, by a combination of complementary in situ techniques, the mechanism of a Cu-Zr-Al metallic glass’s high propensity for metastable phase formation, which is partially through a kinetic mechanism of Al partitioning.

## Introduction

At room temperature, applied strain on metallic glasses (MGs) results in inhomogeneous plastic flow initially localized into ~10–20 nm thick shear bands^1^. Yielding of MGs, with a universal shear strain of 2.67 ± 0.02%^2^, is accompanied by significant softening, resulting in a catastrophic failure. By controlling and tuning intrinsic heterogeneities in MGs^3^ via external stimuli, such as non-affine thermal^4^ and elastic^5^ strains, significant improvement in MGs’ formability is achieved—compressive plastic strain can increase up to ~6%^4^, and MGs can show strain-hardening^6^.

Plasticity can be enhanced by introducing a deformable crystalline phase(s) into the glass^7,8^. For such composites, the mechanical properties can be controlled by tuning the type and size of the crystals formed. For example, in pure nanocrystalline Cu, a bimodal crystal size distribution might be beneficial, with nanometer-sized grains providing strength, combined with micrometer-sized grains giving strain-hardening^9^. The composite formation is better controlled on heating MGs, giving access to a wide range of heating rates (Φ) of about 8 orders of magnitude, rather than on cooling the liquid.

In the archetype equimolar CuZr and derived CuZr-based MGs, the presence of the B2 CuZr phase (space group ${\rm{Pm}}\bar{3}{\rm{m}}$) is beneficial for enhancing plastic compliance of metallic-glass–crystal composites^10^. Some Cu-rich phases give hardness to composites, and they can also provide additional functionalities such as antibacterial properties^11^, though such crystals are brittle and prone to corrosion. The B2 phase is stable at *T* > 900 K, but it can already form on isothermal annealing at 671 K, or during primary crystallization on conventional heating at Φ ≤ 60 K min^−1^ where the crystallization onset temperature, *T*_x_, lies below the B2 forming temperature in the binary system^12–14^. The volume fraction of the B2 phase formed at such conditions is low and B2 decomposes into the equilibrium phases below 900 K^13^.

The B2 phase has been shown to form on fast heating, it can form on a millisecond timescale, translating to Φ in the range of ~10^2^ K s^−1^. Flash-annealing suppresses the formation of the low-temperature thermodynamic equilibrium phases Cu_10_Zr_7_ (space group C2ca) and CuZr_2_ (space group I4/mmm), and gives the possibility to control the B2 phase content. For the Cu_47.5_Zr_47.5_Al_5.0_ MG studied here, Φ as high as ~700 K s^−1^ was suggested to be needed, though Cu_10_Zr_7_ could not be fully suppressed^15^. Composites formed by flash-annealing with Φ >  100 K s^−1^ during resistive (Joule)^15^ and inductive heating^16^ dominated by the metastable B2 phase can achieve a compressive strain of about 10% (crystals: ~10 μm in diameter and ~15 vol.% of B2) for Cu-Zr-based bulk MGs^16^. Flash-annealing may introduce crystals as small as ~1.5–2 nm, which is close to the theoretical limit of the critical nucleus, that can promote crystallization in MGs upon deformation, thereby also improving plasticity when an optimum grain-size range is reached^17,18^. So far, flash-annealing techniques^15,16,19–24^ and ultra-fast differential scanning calorimetry (FDSC)^25–27^, or a combination of both^24^, have been predominantly used to study the kinetics of the glass-transition temperature, *T*_g_, and *T*_x_ at Φ up to ~10^6^ K s^−1^ in Cu-Zr-based MGs. Yet, the crystallization mechanism underlying the observed kinetics could only be accessed ex situ, based on the microstructure and XRD phase analysis, and the existing phase-transformation diagrams have been mostly derived by plotting the projection of CuZr equilibria. The Φ range for in situ X-ray experimental studies of crystallization in Cu_50_Zr_50_ MG, for which details of the mechanism were suggested^12–14^, has been limited to conventional Φ ≤ 60 K min^−1^, which is 2–3 orders of magnitude lower than the Φ required for composite formation. Küchemann and Samwer carried out an in situ X-ray diffraction study of the crystallization sequence during flash-annealing of Zr_64.0_Cu_28.2_Al_7.8_ MG ribbons^19^. However, due to the capacitor-discharge heating, Φ remained limited to high heating rates of ~10^6^ K s^−1^, and the crystallization was dominated by ZrO_2_ formation^19^.

The competition between the formation of the phases needs to be clearly understood to create composites with on-demand and often multifaceted properties, such as has been widely applied to make composites with low-energy-loss soft-magnetic properties^29^. Hitherto, tracking and understanding in situ the phase evolution underlying the fast kinetics was impossible because Φ was not in a region relevant for composite formation, limited by conventional experimental techniques. Therefore, crystallization mechanisms could be deduced from ex situ examinations only, for which, as will be shown later, unwanted phase transformation on cooling may occur. Also, crystallization mechanisms of ternary alloys have been extrapolated from binary systems, especially from Cu_50_Zr_50_ glass, which is a too simplified approach.

In this work, a combination of in situ high-energy X-ray diffraction with a high temporal resolution is carried out during resistive flash-annealing and during containerless solidification via electromagnetic levitation (EML), and those are complemented by in situ transmission electron microscopy (TEM). The crystallization mechanism of the low- and high-temperature phases (represented by Cu_10_Zr_7_, CuZr_2_ and B2 phase, respectively) and kinetics can be correlated in Cu_47.5_Zr_47.5_Al_5.0_ glassy ribbons on ~4 ms timescale. Controlled cooling in a chamber flushed with helium is demonstrated to maximize the B2 content in the composite. The local microstructure is analyzed by atom probe tomography (APT) and TEM; the presence of nanometer-length-scale Al-rich precipitates within and around the B2 phase is resolved.

## Results

### The kinetic conditions of phase transformation

**Fig. 1:**
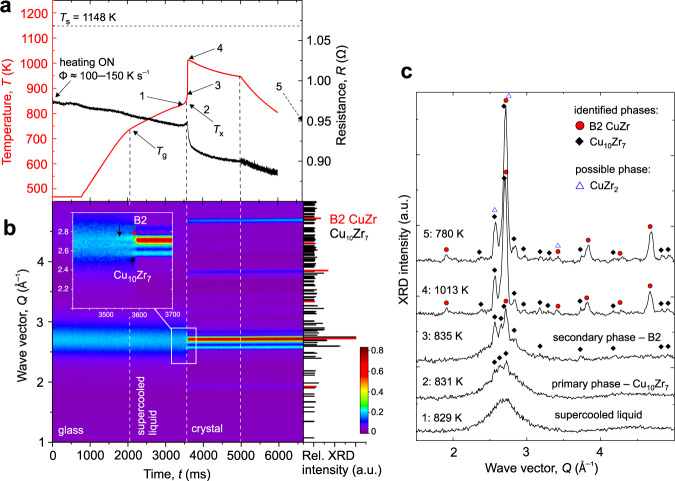
In situ high-energy X-ray diffraction revealing the crystallization mechanism on flash-annealing at a subcritical heating rate of Cu_47.5_Zr_47.5_Al_5.0_ ribbon in a vacuum. **a** Time-dependent temperature (red) and resistance (black) on flash-annealing at Φ ≈ 100–150 K s^−1^. **b** The temporal evolution of high-energy X-ray pattern. The inset shows a close-up of the crystallization onset for Cu_10_Zr_7_ (black arrows) and B2 CuZr (red arrows) phases. The right-hand part in part **b** plots the relative X-ray intensities which are normalized to the highest-intensity peak. **c** Selected individual XRD patterns showing the different stages of crystallization and phase transformations. Full and open symbols label respectively the position of the most pronounced peaks of the identified and possible phases revealed by Rietveld refinement—details can be found in Supplementary Information, Supplementary Fig. 2 (high-energy XRD) and Supplementary Fig. 3 (laboratory XRD). The labeled points correspond to the time of 1: 3505 ms; 2: 3532 ms; 3: 3588 ms; 4: 3610 ms; and 5: 6645 ms. Source data of the XRD patterns are provided on https://archive.materialscloud.org/^61^.

**Fig. 2:**
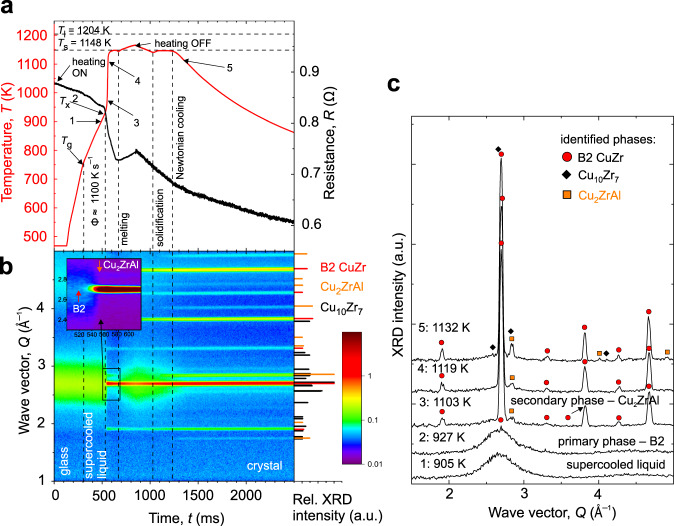
In situ high-energy X-ray diffraction revealing the crystallization mechanism on flash-annealing at a supercritical-heating rate of the glass in a vacuum. **a** Time-dependent temperature (red) and resistance (black) of Cu_47.5_Zr_47.5_Al_5.0_ glassy ribbon when resistively heated at a heating rate of Φ ≈  1100 K s^−1^ and cooled in a vacuum. **b** The corresponding temporal evolution of high-energy X-ray patterns on heating and cooling. The inset, in part **b**, shows a close-up of the crystallization onset for B2 (red arrow) and Cu_2_ZrAl (orange arrow) phases. Right-hand part in part **b**: The relative X-ray intensities are normalized to the highest-intensity peak. For clarity, only selected Cu_10_Zr_7_ diffraction peaks are plotted (see Fig. 1, part **b**, for the complete plot). **c** Selected individual XRD patterns showing the different stages of crystallization and phase transformations. Full symbols label the position of the most pronounced peaks of the identified phases revealed by Rietveld refinement—details can be found in Supplementary Information, Supplementary Fig. 4 (heating), and Supplementary Fig. 6 (cooling). The labeled points correspond to the time of 1: 489 ms; 2: 520 ms; 3: 552 ms; 4: 566 ms; and 5: 1300 ms. Source data of the XRD patterns are provided on https://archive.materialscloud.org/^61^.

A critical Φ to form a composite with a predominant fraction of the B2 CuZr phase in Cu_47.5_Zr_47.5_Al_5.0_ was suggested to be ~700 K s^−1^.^15^ Two flash-annealing examples are presented in detail by in situ XRD: one case shows a subcritical Φ in the range 100–150 K s^−1^ (Fig. 1), and the other a supercritical Φ of about 1100 K s^−1^ (Fig. 2) in a vacuum. The full Φ-dependence is discussed later in the “Mapping the correlation between kinetics and crystallization mechanism” section. For low Φ, the resistive heating is non-linear (Fig. 1a—red curve). All the Φ values given here are estimated by assuming linear heating from room temperature to *T*_x_, and are comparable with flash-annealing^15,30^ and FDSC^28^. It must be noted that according to APT results, the local stoichiometry was reached for the B2 phase only, independent of heating/cooling conditions as detailed in the latter sections. For simplicity, the stoichiometric compound nomenclature is kept throughout the manuscript.

On subcritical heating at 100–150 K s^−1^, *T*_x_ is 831 K represented by the sharp drop in the resistance at *t*_x_ = 3532 ms and the temperature rise on the *T*(*t*)-curve due to recalescence (Fig. 1a). A 3-D representation of the temporal evolution of XRD patterns is given in Supplementary Fig. 1. The following phase identification, and all others discussed in the upcoming sections, is rationalized by Rietveld refinement. For the subcritical Φ, the best-fit refinement is shown in Supplementary Fig. 2. The identified phases at specific stages of heating and cooling are shown in Fig. 1c (only the most pronounced peaks are marked by the corresponding symbols). The crystallization proceeds via the formation of the equilibrium low-temperature Cu_10_Zr_7_ phase preceding the B2 phase formation by ~56 ms (Fig. 1b, c). By analyzing 20 summed intensities taken around point 4 in Fig. 1a, *t* = 3628–3704 ms and *T* ≈  1007–1013 K, the Rietveld refinement confirmed the presence of the two main phases (Supplementary Fig. 2a). The low-temperature CuZr_2_ phase, reported in the literature to form parallel to and/or just after Cu_10_Zr_7_ during the primary crystallization^12–14^, could not be unambiguously identified in the individual high-energy XRD patterns on heating. This is because of CuZr_2_ possible low quantity and the low associated XRD intensity, and because the XRD patterns are predominated by Cu_10_Zr_7_ in the *Q-*range of interest for CuZr_2_. The Rietveld refinement, Supplementary Fig. 2b, hints at the possible presence of CuZr_2_ phase around point 4 in Fig. 1a, but considering high uncertainty of the fitting this just gives an indicative trend, and the exact *T* and *t* for the CuZr_2_ formation on heating and cooling could not be resolved in situ. The possible presence of CuZr_2_ could also be hinted by analyzing laboratory XRD measurements of the final microstructure at room temperature (see Rietveld refinement shown in Supplementary Fig. 3); yet, if anything, the phase fraction must be quite low. When the electrical current ceases at *t* = 5000 ms, the initial cooling rate is ~300 K s^−1^ in a vacuum of 10^−4^ mbar. Due to the low X-ray intensity, there was macroscopically no detectable Cu_10_Zr_7_ coarsening via B2 decomposition as would be expected from the eutectoid reaction of 13CuZr → Cu_10_Zr_7_ + 3CuZr_2_ on cooling Cu_50_Zr_50_^25^; where *T*_e_ = 975 K for the studied glass^28^.

On supercritical heating at ~1100 K s^−1^ (Fig. 2), the Cu_10_Zr_7_ crystallization was suppressed, and B2 became the primary phase at *T*_x_ ≈ 927 K. Because of the higher temperature reached and the suppressed Cu_10_Zr_7_ formation, the high-temperature Cu_2_ZrAl phase (*τ*_4_, space group ${\rm{Fm}}\bar{3}{\rm{m}}$) formed as the secondary phase at *T* ≈ 1103 K delayed by ~32 ms (Fig. 2b, c). The presence of the two main phases on heating was confirmed by Rietveld analysis (Supplementary Fig. 4) of 10 summed intensities around point 4 in Fig. 2a, i.e., between *t* = 552–589 ms corresponding to *T* ≈ 1103–1145 K. It is worth noting that this point is about ~150 K higher than the point 4 shown in Fig. 1a. The ternary phase typically forms on isothermal annealing at temperatures of 1000−1100 K^11,31^, or at *T*_x_ = 861 K on heating Cu_46_Zr_46_Al_8_ glass at 20 K min^−1^^32^. The formation of Cu_2_ZrAl during heating was not considered in a FDSC study of Cu_47.5_Zr_47.5_Al_5.0_ glass as calorimetry does not provide any information required for phase identification^28^. Yet, a DSC trace of heating at Φ  = 1000 K s^−1^ (see Fig. 2a in ref. ^28^) shows a possible two-step crystallization and, by deconvoluting the peak into two gives a second onset temperature for phase formation of ~950 K—this is close to that of ~927 K at Φ ≈ 1100 K s^−1^ in the present work. The values of *T*_g_ ≈ 750 K, *T*_x_ = 831 K at 100–150 K s^−1^ and *T*_g_ ≈ 775 K, *T*_x_ = 927 K at ~1100 K s^−1^ are in good agreement with those obtained by FDSC^28^. This trend also indicates that the linear Φ-approximation for flash-annealing gives a reasonable value for an effective Φ. The fast-heated ribbon could be partially melted while maintaining its shape. When the solidus temperature *T*_s_ = 1148 K was reached, the Cu_2_ZrAl and the B2 phases started to melt; on prolonged annealing, the Cu_2_ZrAl phase melted completely (Fig. 2b, and Supplementary Fig. 3 showing a 3-D representation of the crystallization, re-melting, and the onset of Newtonian cooling). On ceasing the current at *t* = 845 ms, a cooling rate of ~140 K s^−1^ and a small effective supercooling of Δ*T* ≈ 66 K (=*T*_l_ − *T; T*_l_—liquidus temperature) were achieved before the solidification of Cu_2_ZrAl and re-growth of the B2 phase, giving the recalescence at *t* = 1139 ms, took place. With further cooling, Cu_10_Zr_7_ phase formed from the supercooled liquid at *T* = 1132 K and *t* = 1300 ms (Fig. 2c), and this phase formation is generally difficult to suppress (Supplementary Fig. 6). Later, in the TEM and the microstructure-analysis paragraphs, we will show that Cu_10_Zr_7_ coarsens upon cooling in a vacuum; this is also an effect observed for Cu-Zr-based cast glasses^33^. In Fig. 2, the B2 continuous growth was observed on cooling down to *~*1100 K. For the supercritical-heating conditions, the CuZr_2_ phase could not be identified by the phase analysis neither during heating nor during cooling stages (Supplementary Figs. 4 and 6). As in Fig. 1, the similarity in the Bragg reflections makes the CuZr_2_ quantification by XRD difficult, since no distinct peaks were observed we conclude that its fraction must be much lower than for slower Φ shown in Fig. 1. The CuZr_2_ phase will be resolved later by EML.

**Fig. 3:**
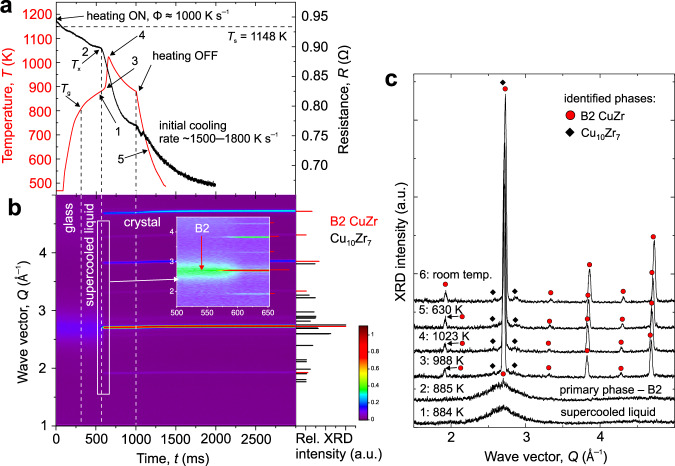
In situ X-ray diffraction revealing crystallization mechanism on flash-annealing in a chamber fluxed with He. **a** Time-dependent temperature (red) and resistance (black) of Cu_47.5_Zr_47.5_Al_5.0_ ribbon when resistively heated at a heating rate of Φ ≈ 1000 K s^−1^ while being fluxed with He. **b** The corresponding temporal evolution of the high-energy X-ray pattern. The inset shows a close-up of the crystallization onset for B2 phase (red arrow). Right-hand part: The relative X-ray intensities are normalized to the highest-intensity peak. For better clarity, only selected Cu_10_Zr_7_ diffraction peaks are plotted. **c** Selected individual XRD patterns showing the different stages of crystallization and phase transformations. Full symbols label the position of the most pronounced peaks of the identified phases revealed by Rietveld refinement—details can be found in Supplementary Information, Supplementary Fig. 7. The labeled points correspond to the time of 1: 541 ms; 2: 545 ms; 3: 606 ms; 4: 657 ms; 5: 1165 ms, and 6: 7468 ms (room temperature). Source data of the XRD patterns are provided on https://archive.materialscloud.org/^61^.

The above results suggest that there is the formation of the brittle low-temperature phases, predominantly Cu_10_Zr_7_ and possibly minor CuZr_2_, on cooling in vacuum. To suppress this undesirable formation and to better control the B2 phase fraction, a chamber flushed with He giving cooling rates 1000–2000 K s^−1^, one order of magnitude higher than in a vacuum, was used. In Fig. 3, about two times larger electric power was applied to compensate for the efficient cooling to achieve Φ comparable with flash-annealing in a vacuum. The highest temperature reached was 1023 K (Fig. 3a), ~50 K above *T*_e_. The primary crystallization was dominated by the B2 phase formation at *T*_x_ ≈ 885 K, *t*_x_ = 545 ms (Fig. 3c). Defining one average Φ, unlike in vacuum, is rather too simplified in the case of He atmosphere. When the glass enters its supercooled liquid region, the efficient He cooling results in effectively much slower Φ (see the slowing down in the effective Φ in Fig. 3a). This provided some time for the appearance of the sluggish Cu_10_Zr_7_ phase, yet its fraction was extremely low, and it could only be detected at later stages of heating, when a larger volume fraction had formed (see Supplementary Fig. 7 showing Rietveld analysis at point 4 of the heating part in Fig. 3a; 20 individual XRD patters were summed between *t* = 609–686 ms corresponding to *T* ≈ 978–1024 K). There was no evidence for the presence of CuZr_2_ and Cu_2_ZrAl phases in the XRD pattern by the Rietveld refinement. On cooling, unlike in Fig. 2, there was no detectable Cu_10_Zr_7_ coarsening (see also Supplementary Fig. 8 for a 3-D representation).

**Fig. 4:**
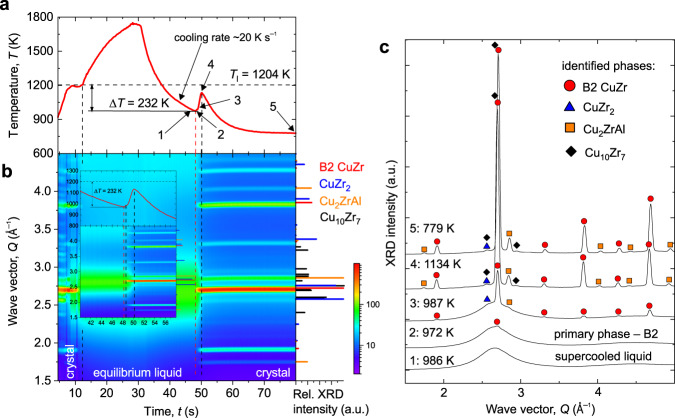
In situ high-energy X-ray diffraction showing phase-formation sequence during solidification of supercooled Cu_47.5_Zr_47.5_Al_5.0_ liquid in EML facility. **a** Temperature profile showing the achieved supercooling of Δ*T* = 232 K = (*T*_l_ − *T*); *T*_l_ is the liquidus temperature. A typical cooling rate before the recalescence is ~20 K s^−1^. **b** The corresponding evolution of X-ray pattern measured at 5 Hz. The inset shows a close-up of the solidification process. Right-hand part: The relative XRD intensities of selected peaks normalized to the highest-intensity peak are depicted. **c** Selected individual XRD patterns showing the different stages of solidification on cooling. Full symbols label the position of the most pronounced peaks of the identified phases revealed by Rietveld refinement—details can be found in Supplementary Information, Supplementary Fig. 9. The labeled points correspond to the time of 1: 47 s; 2: 48.2 s; 3: 48.6 s; 4: 50.2 s; and 5: 80 s. Source data of the XRD patterns are provided on https://archive.materialscloud.org/^61^.

In order to understand the details of the solidification of Cu_47.5_Zr_47.5_Al_5.0_ supercooled liquid and the competition in phase formation on cooling, containerless processing^34^ via EML (Fig. 4) was used to complement the flash-annealing. The XRD instability seen in Fig. 4b during heating up to *T*_s_ was caused by sample oscillations (shape instabilities) whilst levitating the solid-state alloy inside a Cu-coil, and extra peaks belonging to the Al_2_O_3_ sample support were detected. Heating was used to determine *T*_l_ for pyrometer calibration only. The liquid supercooling Δ*T* was calculated from the lowest *T*-point read by the pyrometer before a steep temperature rise occurred due to the recalescence. Along the lines of thoughts for flash-annealing, Rietveld analysis of the XRD intensity, taken at point 4 in Fig. 4a, *t* = 50.2 s and *T* = 1134 K, was carried out. The presence of all phases being relevant in this study, i.e., B2 CuZr, Cu_10_Zr_7_, CuZr_2_, and Cu_2_ZrAl can be seen in Supplementary Fig. 9. On cooling, the B2 always nucleated as the primary phase—the kinetics is evaluated in the next section. In the experiment presented in Fig. 4, B2 phase formed at an apparent nucleation temperature of *T*_n_ = 972 K (Δ*T* = 232 K), followed by Cu_2_ZrAl and CuZr_2_ phases both forming at *T* ~ 987 K and delayed by 0.4 s relative to the appearance of B2; where the delay time Δ*t* = *t*_x_ − *t*_l_; *t*_l_ = 0 s when *T*_l_ is passed on cooling. For some supercooling conditions, weak Bragg reflections of the B2 phase were identified before the recalescence event resolved by pyrometer, a possible consequence of the stochastic nature of the nucleation. Although the CuZr_2_ phase could not be unambiguously identified in flash-annealing, the complementary data by EML readily evidence this phase, and that the overall phase-transformation mechanism conforms to the flash-annealing results. The Cu_10_Zr_7_ phase possibly nucleated during the recalescence, but it could only be identified on XRD intensity beginning from point 4, shown in Fig. 4a, c, when enough volume fraction had formed.

**Fig. 5:**
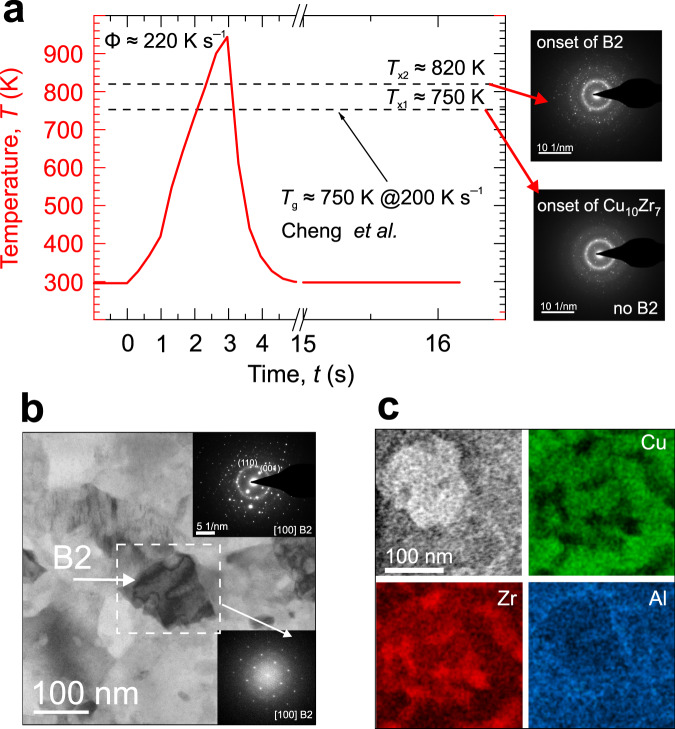
In situ TEM showing the crystallization mechanism and the final microstructure formed in a confined environment at a subcritical heating rate of about 220 K s^−1^ in a vacuum. **a** Heating profile of a glassy Cu_47.5_Zr_47.5_Al_5.0_ thin foil. Two crystallization temperatures *T*_x1_ and *T*_x2_ corresponding respectively to the onset of Cu_10_Zr_7_ and B2 phase formation—represented on the right-hand by SAED patterns—can be identified. The value of the glass-transition temperature, *T*_g_, of a ribbon sample is taken from Cheng et al.^28^ and was obtained by ultra-fast scanning calorimetry. The maximum temperature reached on heating was *T*_max_ ≈ 944 K, followed by cooling at a rate of ~1090 K s^−1^. The phase transformation was confined to a volume of ~16 × 0.05 μm^3^ (area × thickness). **b** Bright-field STEM micrograph and SAED (inset in the top-right corner) of the final microstructure. The white rectangle highlights a B2 grain surrounded mostly by Cu_10_Zr_7_, and it also marks the area of the corresponding fast-Fourier-transform pattern shown as the inset in the bottom-right corner. **c** HAADF image and the corresponding EDX of the individual elements (Cu: green; Zr: red; and Al: blue) of the area in the B2 crystal vicinity from part **b**.

Complementing the in situ XRD studies above, heating inside TEM was used to understand local conditions under which the individual phases form on heating and on cooling (Fig. 5). Local amorphicity in the as-spun ribbon, without indications of clustering and phase separation, was confirmed by HRTEM and SAED (Supplementary Fig. 10). The composition by EDX was Cu_48_Zr_47_Al_5_ (±1 at.%). A top oxide layer, ~5 nm thick, had no observable influence on the annealing experiments. No thermal drift was observed^35^. Heating of a thin foil at Φ ≈ 220 K s^−1^, a Φ-range dominated by Cu_10_Zr_7_ formation, revealed a distinct two-step crystallization with *T*_x1_ = 750 K (*t*_x1_ = 2.06 s) and *T*_x2_ = 820 K (*t*_x2_ = 2.35 s) corresponding to Cu_10_Zr_7_ and the B2 phases (Fig. 5a—SAED patterns), respectively. Faster heating could not be achieved inside the TEM. Once the Cu_10_Zr_7_ phase had appeared, it did not grow, unlike the B2 phase, which coarsened until ~944 K. The presence of two minor phases could be distinguished from the SAED patterns: (i) CuZr_2_—mostly detected at the initial stages of heating; its appearance agrees with the possible presence of CuZr_2_ during subcritical Φ (Fig. 1), although at different stages. It was not possible to detect any time delay between the Cu_10_Zr_7_ and CuZr_2_ formation in TEM, which conforms to the conventional in situ XRD study of Cu_50_Zr_50_ glass at a heating rate of 10 K min^−1^ by Kalay et al.^13^. (ii) Cu_2_ZrAl—detected at the cooling onset. On cooling, predominantly Cu_10_Zr_7_ phase coarsening without noticeable B2 growth was observed. The Cu_10_Zr_7_ coarsening is evidenced in Supplementary Fig. 11, which shows the integrated temporal evolution of the SAED patterns. Because of the confined crystallization, *T*_x1_ is suppressed by about 100 K compared to a ribbon^28^, and the delay of ~0.3 s in the B2 formation is about one order of magnitude longer than for flash-annealing (Fig. 1). A non-monotonic size-dependent crystallization, in terms of *T*_x_ and phase selection, was also observed for a sandwiched thin-film Cu-Zr^36^ and a Pt-based MG in a form of nanorods with diameters of <200 nm^37^.

### Mapping the correlation between kinetics and crystallization mechanism

**Fig. 6:**
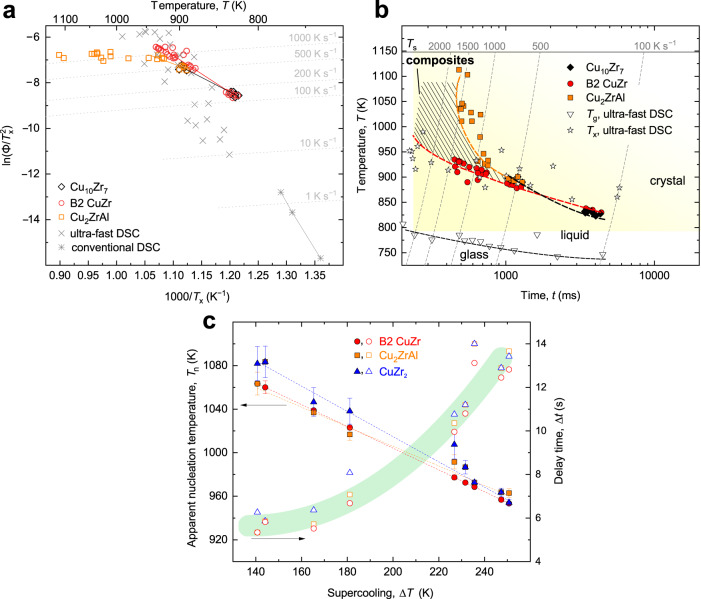
Correlating the kinetics and phase-transformation mechanism in metallic-glass Cu_47.5_Zr_47.5_Al_5.0_. **a** A Kissinger plot for the crystallization of the individual phases during flash-annealing. The CuZr_2_ phase is not plotted because it could not be unambiguously resolved during flash-annealing. The apparent activation energies for crystallization are shown by the solid lines representing best-fits by assuming Arrhenius kinetics. The ultra-fast-heating (FDSC) and conventional calorimetry data for *T*_x_ are taken from Cheng et al.^28^ The dashed contours show the nominal heating rates. **b** Continuous-heating-transformation phase diagram for flash-annealing (present work) and for ultra-fast differential scanning calorimetry taken from the literature^28^. Only *T*_g_ obtained by FDSC is shown as this can be measured more accurately than by flash-annealing. The plotted nominal heating rates in K s^−1^ are based on values obtained from FDSC. The range of temperature and heating rate under which ductile composites can be formed during flash-annealing is highlighted by the hatched area. **c** The dependence of the apparent nucleation temperature, *T*_n_ (left-hand axis—full symbols) and the delay time, Δ*t –* defined as 0 s at *T*_l_ – (right-hand axis – open symbols) on supercooling (Δ*T* = *T*_l_ − *T*) obtained by containerless solidification via electromagnetic levitation. The dashed lines show linear best-fits to the experimental data. For *T*_n_ and Δ*t*_n_ at large Δ*T*, the error bars are smaller than the symbols size.

The comprehensive in situ evaluation of the kinetics presented above can be summarized and represented by a Kissinger plot^38^ (Fig. 6a) and by a continuous-heating-transformation (CHT) diagram (Fig. 6b) for crystallization—which can be correlated with the phase-transformation mechanisms given above—and by the dependence of *T*_n_, and the delay time, Δ*t*, on Δ*T* for levitation (Fig. 6c). *T*_x_ is taken from the first XRD pattern when a given phase is detected, which, for a primary phase, typically lies slightly before a recalescence event. Independent of the crystallization mechanism, any present-work primary phase formed superimposes on the overall kinetics by FDSC^28^ at a given Φ (Fig. 6a, b); some discrepancy arises due to Φ approximations. Unlike calorimetry measurements, the kinetics of the individual phases can readily be resolved. A Kissinger plot^38^ reflects the temperature-dependent activation energy for crystallization^39^ and by taking the Arrhenius parts it gives *E*_a_(B2) = 134 ± 5 kJ mol^−1^; *E*_a_(Cu_10_Zr_7_) = 106 ± 4 kJ mol^−1^; and *E*_a_(Cu_2_ZrAl) = 75 ± 12 kJ mol^−1^ (*T* < 950 K); where *E*_a_ = 342 ± 8 kJ mol^−1^ by taking the conventional DSC only. For Φ < 500 K s^−1^, Cu_10_Zr_7_ is the primary phase, and for Φ > 500 K s^−1^ and higher, the low-temperature phase is suppressed and B2 becomes the primary phase up to the measured ~1500 K s^−1^ and likely extends up to the critical heating rate of ~10,000 K s^−1^ beyond which crystallization is avoided^28^. The lowest *T*_x_ at which Cu_2_ZrAl was detected was ~890 K; the *E*_a_ leveling off after 950 K (Fig. 6a) and the nose at ~1050 K at ~1500 K s^−1^ in CHT (Fig. 6b) suggest that the phase may have reached its maximum transformation rate^39^ and it becomes kinetically suppressed on approaching *T*_s_. For EML, the solidification is nucleation-dominated for all phases that formed from the supercooled liquid. The solidification mechanism was nearly independent of Δ*T* in the range 141–251 K, where the latter corresponds to 953 K (0.83*T*_s_) which lies below the expected maximum in the polymorphic crystal-growth rate at ~1000 K^40,41^. There was a tendency for Cu_2_ZrAl to nucleate before the CuZr_2_ phase at smaller Δ*T*, whereas they formed in parallel at larger Δ*T*. The largest Δ*T*, a relative Δ*T*/*T*_l_ = 0.21, is smaller than that achieved for Cu_50_Zr_50_ liquid via electrostatic levitation^42^.

### Nanostructure and composition of the transformed systems

The in situ TEM, Fig. 5, showed that the final microstructure remained partially amorphous (SAED in Fig. 5b, top-right corner; and see also Supplementary Fig. 12 in Supplementary Information which shows an HRTEM image from one of the largest amorphous regions and the corresponding SAED pattern), and these regions probably separate the B2 crystals from Cu_10_Zr_7_ grains. A B2 crystal, oriented close to the [100] zone axis, is in the center of Fig. 5b (marked by the rectangle) and is surrounded by Cu_10_Zr_7_ crystals. EDX of the CuZr crystal confirmed a 50.5:49.5 (±0.5 at.%) ratio. There were some regions with a higher atomic fraction of Zr in comparison with Cu, yet, their origin remained unclear and they seemed to spatially correlate with the amorphous regions. Most notably, precipitation of Al around the B2 crystal could be detected (Fig. 5c, bottom-right image).

**Fig. 7:**
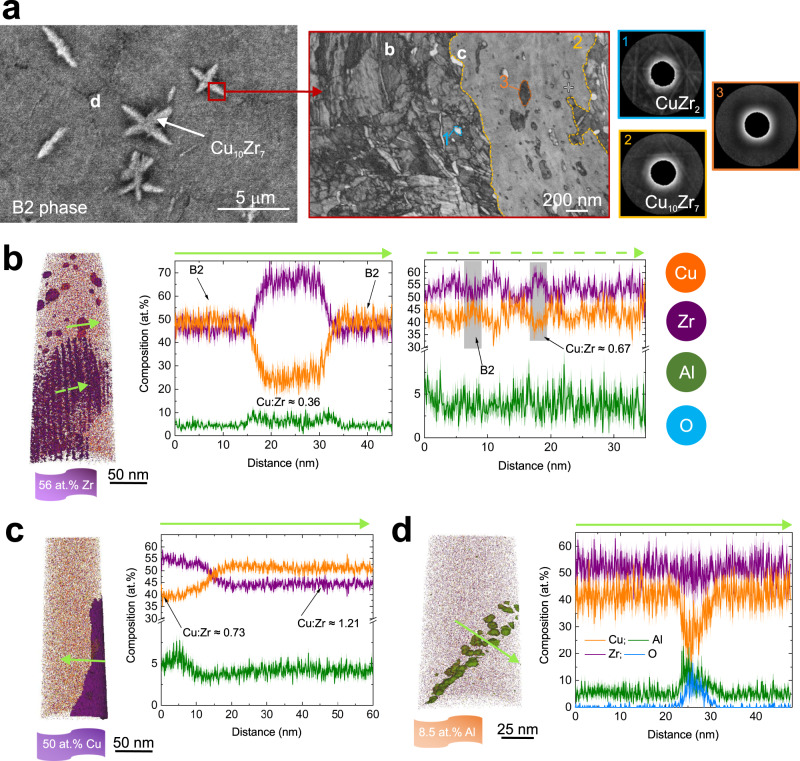
Atom probe tomography reconstructions of the local microstructure after flash-annealing at a supercritical-heating rate as shown in Fig. 2. **a** Left: SEM micrograph of an area from which APT samples were extracted. Middle: Transmission Kikuchi diffraction image quality map showing details of the interface between Cu_10_Zr_7_ (the region enclosed by the yellow borderline) and B2 phases (uncolored); the latter contains CuZr_2_ phase (the region enclosed by the blue borderline). Right: Individual Kikuchi patterns taken from the mapping depicted in the middle (blue: CuZr_2_, yellow: Cu_10_Zr_7_, orange: amorphous). The letters in the left and the middle micrographs mark different regions where the APT samples illustrated in **b**–**d** were extracted from. **b** 3-D atom map showing precipitates within the B2 matrix and a local eutectoid microstructure consisting of linear features arranged as a plane with the corresponding 1-D concentration profiles. **c** 3-D atom map of a region dominated by Cu_10_Zr_7_ phases is shown together with the corresponding 1-D concentration profile. **d** Aluminum precipitation within the B2 crystal and the corresponding 1-D concentration profile.

A representative SEM micrograph of a polycrystalline sample after flash-annealing in vacuum shows dendrites of Cu_10_Zr_7_ phase surrounded by spheroidal grains of B2 (Fig. 7a; a lower-magnification micrograph is presented in Supplementary Fig. 13). The surface density of nuclei, *N*_s_, calculated from a high-speed video taken in situ by camera^30^ was nearly constant ~(0.2–1.2) × 10^5^ m^−2^ (Supplementary Fig. 14) in the range of 50–950 K s^−1^. Similar independence of *N*_s_ on isothermal annealing temperature was observed in oxide-based glass-forming liquids^43^. The volume nuclei density decreased from 1.23 × 10^17^ to 2.63 × 10^16^ m^−3^ with increasing Φ (Supplementary Fig. 14). Although this contrasts with the usual trend, at least within the range of conventional Φ, similar *N*_v_–Φ dependence was reported for flash-annealed Cu_44_Zr_44_Al_8_Hf_2_Co_2_ bulk MG by Kosiba et al.^16^, who suggested a lower effective nucleation rate during flash-annealing than for steady-state conditions.

Based on the APT analysis, no chemical heterogeneities were detected in the as-spun ribbon. The bulk composition of Cu_46.5_Zr_47.0_Al_5.3_ (Supplementary Fig. 15) was measured which is close to nominal. The difference of ~1.5 at.% can be attributed to measurement errors and to the presence of trace elements such as oxygen and hydrogen. APT samples of the flash-annealed glasses were taken from the vicinity of the Cu_10_Zr_7_/B2 interface by site-specific FIB lift-out preparation. The locations are labeled by the corresponding letters and presented in Fig. 7, parts (b), (c), and (d) for vacuum-heated glasses. A supercritical-heating rate of ~900 K s^−1^ to predominantly from the B2 phase was used, which conforms to the thermal history shown in Fig. 2 without the melting step. The maximum temperature was 1050 K, ~75 K above *T*_e_. Local Kikuchi mapping showed that some parts remained amorphous (Fig. 7a—orange Kikuchi pattern), which conforms to the TEM observations (Fig. 5b and Supplementary Fig. 12). Some regions of the material contained unindexed crystallization phases because mapping with extremely low confidence indexing was excluded. The yellow region highlights the indexed area of a macroscopic Cu_10_Zr_7_ region surrounded by the B2 phase (uncolored). A small amount of CuZr_2_ phase was identified—highlighted in blue (Fig. 7a). Different microstructural features were identified in APT reconstructions. The APT sample matrix consisted of the B2 phase with a composition close to 1:1 Cu:Zr ratio (Fig. 7b; see Supplementary Fig. 16 for chemical analysis). There were two kinds of Zr-rich precipitates in the B2 matrix: spheroidal particles of up to ~20 nm in diameter with the atomic ratio Cu:Zr ≈ 0.36 (Fig. 7b) and, in some samples, eutectoid-like structures (lamella thickness of ~5 nm) consisting of lamellae with nearly equiatomic CuZr composition and with Cu:Zr ≈ 0.67 ratio. The latter ratio represents some transient (metastable) composition, frozen-in states on the way to the equilibrium phases, and it is close to that in Cu_5_Zr_8_ (Cu:Zr = 0.63) whose existence in the binary glass is still in dispute in the literature^13^. Also, Zr-rich precipitates with Cu:Zr ≈ 0.73 ratio could be identified located at the interface between B2 phase and Cu_10_Zr_7_ dendrites (Fig. 7c). The Cu:Zr atomic ratio in the Cu-rich phase at the interface was ≈1.21, which corresponds to slight Cu-deficiency of the nominal Cu_10_Zr_7_ (Cu:Zr = 1.43) phase. All of these reveal a possible tendency towards the formation of CuZr_2_ phase along with Cu_10_Zr_7_ phase. Interestingly, Al-enriched precipitates with a diameter of <10 nm, containing up to ~12 at.% of Al, were typically located along the internal boundaries within the B2 phase (Fig. 7d). The local Al precipitation conforms to that revealed by TEM around the B2 crystal (Fig. 5c).

A ZrO_*x*_ layer (Supplementary Fig. 17), ~20 nm thick, was found at the interface shown in Fig. 7c, while no macroscopic oxides were observed by XRD, though Cu-Zr-based MGs are prone to oxidation^44^. A detailed study of the surface conditions by Auger and XPS analysis found that for the pristine surface the Zr3d binding energy revealed Zr in the form of an oxide and Cu2p was metallic. Down to a depth of a few nanometers, Zr3d existed in two oxidation states as an oxide and metal, while Cu2p remained metallic (Supplementary Figs. 18 and 19 showing Auger and XPS spectra, respectively). The oxide thickness decreased from ~15 to ~10 nm as Φ increased. The glass properties are oxygen-sensitive—higher oxygen content may have a strong influence on the balance between surface and volume crystallization^43^; it reduces the glass-forming ability and suppresses the B2 phase growth^45^, therefore higher critical heating rate has to be applied to obtain predominant B2 phase for glasses with a higher content of oxygen.

**Fig. 8:**
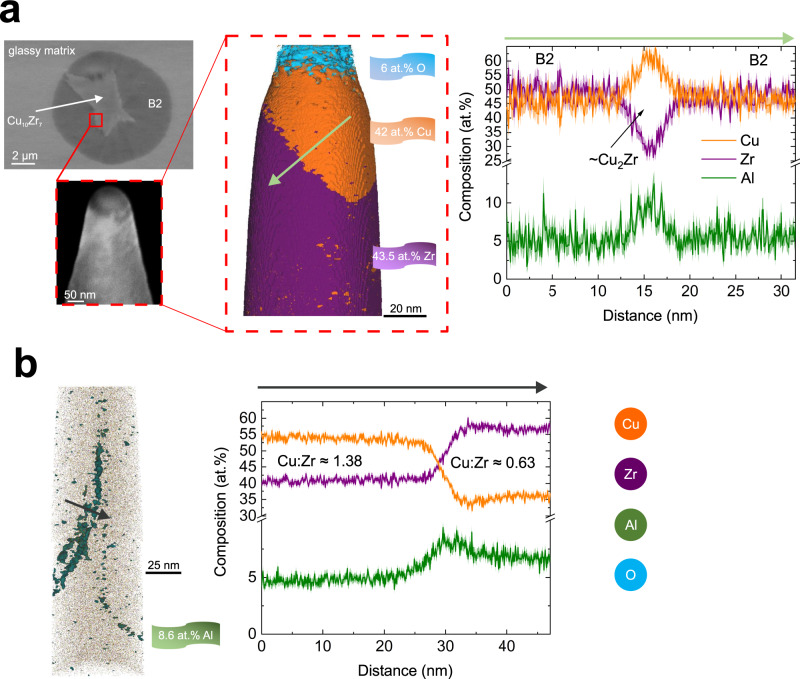
Atom probe tomography reconstructions of the local microstructure of a metallic-glass-crystal composite formed in a chamber fluxed with He. **a** Left: SEM micrographs showing the B2 (spheroid)/Cu_10_Zr_7_ (dendrite) interface from which an APT sample was prepared. Middle: APT volume rendering of the interface. Right: The corresponding 1-D concentration profile. **b** APT volume rendering of Al from a different region and showing the precipitation.

Representative SEM micrographs of glasses heated under helium are shown in Fig. 8a and Supplementary Fig. 20. APT samples were taken from the interface between the dendritic and spheroidal grains (Fig. 8a). APT reconstruction of a pure B2 phase confirmed the nearly equimolar composition (Fig. 8a—the B2 matrix) conforming to vacuum heating. Detailed analysis of the interface found a compact oxide layer of ZrO_*x*_, ~20 nm thick (Fig. 8a), which was more pronounced than in the vacuum-heated ribbons. Auger spectroscopy confirmed the oxide layer of <20 nm thick (Supplementary Fig. 21). The chemical nature of Zr and Cu conformed to those before and after sputtering found during vacuum heating (Supplementary Fig. 19). Below the oxide layer, Cu-rich and Zr-rich phases were found. The former phase atomic ratio was Cu:Zr ≈ 1.38, which is close to that of Cu_10_Zr_7_, and the latter was Cu:Zr ≈ 0.63, resembling, as in a vacuum, the ratio in the speculated Cu_5_Zr_8_ phase (Fig. 8b)^13^. Because of the complex, non-planar interface morphology, different compositions could be mapped including one suggestive of the high-temperature Cu_2_Zr phase, <5 nm thick (Fig. 8a). As for vacuum heating, local Al-enriched precipitates, ~10 at.% and less of Al could be found in the B2 matrix (Fig. 8a).

## Discussion

In situ XRD under isothermal annealing (*T* = 671 K, *t* = 37 min)^12^ and at conventional^13^ Φ = 10 K min^−1^ of the equimolar Cu_50_Zr_50_ glass, often used as the prototype crystallization mechanism, suggested that fine Cu_10_Zr_7_ particles are formed during primary crystallization; on prolonged annealing Cu_10_Zr_7_ grows, accompanied by solute partitioning leading to Zr enrichment in the glass. This is followed by the appearance of CuZr_2_, which is quickly terminated when <20 nm thick, and which already templates the B2 phase growth during primary crystallization. The B2 then dissolves and re-precipitates at 1002 K on heating^13^.

Here, we provide direct experimental evidence that the crystallization mechanism originally suggested for the equimolar binary glass can only be extended to ternary systems and to higher Φ with caution. The proposed templated-growth mechanism of the B2 phase could not be clearly resolved, although, especially fur subcritical Φ it is reasonable to suggest that the dendrites are located at the center of B2 grains (Supplementary Fig. 13) and Cu_10_Zr_7_ particles could act as nuclei for the B2 phase. For flash-annealing, all phases formed directly from the supercooled liquid, there were no detectable solid-state transformations, and no B2 re-precipitation could be observed. The B2 phase did not undergo any detectable martensitic transition to the previously reported B19’ and B33 two-phase structure^13,46^. The in situ XRD (Figs. 1, 2, for vacuum and Fig. 3 for He-atmosphere heating) did not reveal any eutectoid reaction of Cu_10_Zr_7_ + 3CuZr_2_ → 13CuZr; only local eutectoid-like microstructure could be revealed in some samples by APT. As shown in Fig. 7b, a local fine-scale eutectoid microstructure of alternating B2 and Zr-rich phases can exist, the latter probably locally compensating for the formation of Cu_10_Zr_7_ phase; also, local variations in the composition and cooling rates may exist. Arguably, both flash-annealing XRD and TEM (for the latter a strong phase selection due to nanoconfined crystallization cannot be ruled out) only hint at the presence of CuZr_2_ phase during heating/cooling. The absence of the CuZr_2_ phase on heating would contradict highly sensitive calorimetry experiments where the eutectoid reaction in a thin-film equimolar CuZr glass could be resolved even on fast heating (Φ ≈ 21,000 K s^−1^)^25,47^. On the other hand, the APT reconstructions revealed the presence of a different Zr-rich phase, where the Cu:Zr ratio resembled that of Cu_5_Zr_8_^13^. The compositions from APT should not be simplified by using binary chemistry only. Clearly, Al is always present in the quoted phases, but no tendency to phase separation in the glass, as in an Al-rich MG^48^, and no composition resembling, for example, that of Cu_2_ZrAl in the composite were found. There was a clear tendency to partition Al from the B2 phase, but not from the other phases: an Al-enrichment, with thickness <10 nm, around the nanoconfined B2 phase boundary was found by TEM (Fig. 5c); and Al-enriched precipitates, with diameter less than ~10 nm, containing up to ~12 at.%, were typically observed along the internal boundaries within the B2 spheroid by APT (Figs. 7d and 8a). Generally, Al helps to improve the glass-forming ability^49^, and its partitioning supports the fast polymorphic growth of the B2 phase. Also, aluminum is highly mobile; the self-diffusion coefficient is 7.2 × 10^−9^ m^2^ s^−1^ at *T* = 980 K^50^, and this is high enough to permit strain-induced intermixing^51^ and the formation of pure Al nanocrystals^52^ inside shear bands. Interestingly, for the equimolar binary glass, recent APT work showed a tendency to the formation of the equilibrium Cu_10_Zr_7_ and CuZr_2_ phases in shear bands on deformation at room and nominal 80 K temperature^53^.

Under what conditions can ductile composites be made? A critical Φ relevant for the composites is ~500 K s^−1^ and higher (Fig. 6b). Below that value, Cu_10_Zr_7_ precedes the B2-phase formation. B2 becomes the primary and dominant phase followed by Cu_2_ZrAl at a higher temperature, whose formation is quickly suppressed beyond ~1500 K s^−1^. Although Cu_10_Zr_7_ could not be detected at high Φ, probably because of low X-ray sensitivity, it may be that some nuclei of Cu_10_Zr_7_ may still be present, whether existing predominantly on the surface or in the volume remains unclear, which can grow on consequent cooling. To obtain composites with a maximized fraction of the B2 phase on heating, a minimum Φ of ~1500 K s^−1^ should be applied, giving *T*_x_ ~930 K. Such a high Φ gives a relatively narrow temperature range of ~200 K for which the upper-limiting temperature can be controlled before melting is reached. The other way to control the composite formation is by flash-annealing in a chamber flushed with helium. The efficient cooling gives much better control of the upper-limiting temperature, suppresses the possible unwanted phase transformation on cooling, and gives composites with a lower fraction of Cu_10_Zr_7_, though its formation could not be fully avoided. No local eutectoid-like microstructure could be found under He atmosphere, and it is known that when B2 phase is cooled sufficiently fast such decomposition can be avoided or suppressed in the binary MG^47^. The helium atmosphere helps to avoid the temperature overshoot around *T*_x_ which would quickly result in fully crystalline samples.

The high propensity of the glass for the B2 phase formation seems to stem from the following factors: (i) kinetics—higher Φ promotes polymorphic crystallization; (ii) chemistry—local Al-enrichments are induced on crystallization resulting in having the B2 phase close to 50:50 ratio, i.e., the only composition corresponding to the equilibrium to be found among the crystallization products; (iii) atomic packing—there is effectively no difference in the number density between CuZr B2 crystal (0.0576 Å^−3^) and Cu_47.5_Zr_47.5_Al_5.0_ glass (0.0577 Å^−3^), while the number density of Cu_10_Zr_7_ (0.0609 Å^−3^) and CuZr_2_ (0.0518 Å^−3^) phases is, respectively, ≈5.5% and ≈10.2% smaller^54^. Therefore, the formation of Cu_10_Zr_7_ and/or CuZr_2_ phase in the glassy matrix may result in significant raise of local stress; and (iv) atomic structure—there is a close resemblance for the Cu-Zr atomic pairs in the first-coordination shell of the glass and the B2 phase as shown in the structural study elsewhere^54^. Interestingly, composites containing the nanocrystalline B2 phase can be obtained through thermal cycling of the ternary glass between room and liquid-nitrogen temperature^55^—supporting the high propensity of the glass for the B2 phase formation.

To conclude, the mechanism of crystallization and solidification could be correlated in situ with phase-transformation kinetics and related to the formation of ductile metallic-glass─crystal composites on flash-annealing of Cu_47.5_Zr_47.5_Al_5.0_ glass. A complex CHT diagram was directly obtained, unlike the former projections based on CuZr phase equilibria. The primary onset parameters, namely crystallization time and temperature, clearly correlate with the former kinetics measurements obtained via ultra-fast-heating calorimetry. For calorimetry, the kinetics could not be understood in terms of the crystallization mechanism of individual phases and no high-temperature phase transformations following the primary crystallization could be detected. The B2 phase, beneficial for plasticity, becomes the primary crystallization phase when Φ is 500 K s^−1^ and higher. Such a critical rate exceeds that for the low-temperature phase Cu_10_Zr_7_ to be kinetically suppressed, though it cannot be fully avoided in the final composites. EML evidenced the presence of the CuZr_2_ phase, which can be expected on fast heating, but it could not be unambiguously resolved in the high-energy X-ray patterns of flash-annealing, by in situ TEM or by APT. On the other hand, higher rates of ~1500 K s^−1^ are needed to get better control of the crystallization sequence upon which the brittle high-temperature Cu_2_ZrAl phase is suppressed. The lowest temperature at which Cu_2_ZrAl formed was ~890 K. So, both the low- and the high-temperature phases need to be suppressed on heating. The eutectoid crystallization mechanism suggested for the prototype binary Cu_50_Zr_50_ glass was not identified by the in situ XRD, but a local nanometer-length-scale eutectoid structure consisting of alternating B2 and Zr-rich phase was identified by APT. Because of the unwanted Cu_10_Zr_7_ formation on cooling, the B2 fraction can be enhanced by employing heating in He atmosphere to achieve a cooling rate of 1000–2000 K s^−1^.

One way the glass increases its propensity for B2 phase formation is by partitioning Al from the growing crystals to form locally Al-enriched precipitates, <10 nm in diameter, and containing up to 12 at.% of Al. Such a kinetic mechanism of Al precipitation is still pronounced even when fast cooling is achieved in a chamber flushed with helium. Because of highly non-equilibrium conditions of flash-annealing, mainly metastable compositions were detected. The multi-technique approach used in this work can be applied to map phase transformations in other metallic-glass-forming systems and to understand the phase-transformation kinetics in order to control processing parameters to form composites with enhanced ductility.

## Methods

### Sample preparation

A pre-alloy of Cu_47.5_Zr_47.5_Al_5.0_ (at.%) was prepared from high-purity elements (≥99.99%) by arc-melting in Ti-gettered Ar-atmosphere. Each ingot was re-melted three times to ensure chemical homogeneity. The ribbon (~30 µm thick and ~2 mm wide) was melt-spun from the master alloy at *T* ≈ 1320 K on a Cu wheel rotating with a peripheral speed of 29 m s^−1^.

### Flash-annealing and in situ synchrotron experimental setups

Details of the constant-current resistive-heating device and its implementation into a high-energy X-ray beam at DESY, Hamburg, are given in ref. ^30^ Ribbons were resistively heated in a constant-current mode by using a Delta Elektronika SM 52-30 power supply. Ribbon surface temperature was monitored by using a LumaSense IGA 320/23-LO pyrometer with a temporal resolution of 2 ms (in analog mode) and calibrated on the known melting point of the glass. All signals were acquired by a National Instruments USB-6211 acquisition card—data were collected at a sampling rate of up to 48 kS s^−1^. The typical error in the measured resistance was <0.5%. All heating experiments were carried out in transmission mode either in a vacuum of 10^−4^ mbar or in a chamber flushed with helium (6 N) at 5 cm^3^ min^−1^ to control cooling. High-energy X-ray diffraction was done in situ at the P21.1 beamline (wavelength 0.1204 Å, beam size ∼900 × 900 μm^2^). Scattered intensities were captured using a hybrid pixel Dectris PILATUS3 X CdTe 2 M detector (pixel size 172 × 172 µm^2^) operated at 250 Hz. The sample-to-detector distance was 1021 mm for vacuum heating (Figs. 1 and 2) and 1412 mm for heating under He (Fig. 3). Diffractograms were derived from the recorded 2-D patterns by azimuthal integration using the Fit2D v17.006 software^56–58^. The diffraction patterns of individual phases are taken as: ICDD PDF number 00-047-1028 for Cu_10_Zr_7_ phase; ICSD collection code 103163 for B2 phase; ICSD collection code 656062 for Cu_2_ZrAl phase; and ICSD collection code 151846 for CuZr_2_ phase. Rietveld refinement was done in HighScore Plus^59^ 4.7a (built 4.7.1.25061) by using ICCD database versions PDF-4 + 2019 and PDF-4 + 2020.

### Electromagnetic levitation

Details of the EML device used for the measurements can be found in ref. ^60^ Metallic-glass samples of weight 0.5–0.7 g were used. The EML chamber was initially evacuated to 5 × 10^−5^ mbar and then re-filled with a high-purity He gas up to a pressure of 500–700 mbar. Glass heating and positioning were controlled via a water-cooled copper coil powered by a 10-kW generator operating at 280 kHz; the sample was cooled by recirculating the inert gas across the sample with a flow of up to 28 l min^−1^; while maintaining levitation, samples could be cooled down to ~780 K. The temperature was monitored by a pyrometer, implemented in a LASCON Process Controller LPC03 unit, with a temporal resolution of 20 ms and this was calibrated on the known liquidus temperature of the alloy, *T*_l_ = 1204 K.

The high-energy X-ray scattering experiments were carried out in transmission geometry, the beam energy was 101.537 keV (corresponding to a wavelength of 0.1221 Å), and data were collected using a hybrid pixel Dectris PILATUS3 X CdTe 2 M detector (pixel size 172 × 172 µm^2^) operated at 5–50 Hz. The sample-to-detector distance was 1100 mm. Diffractograms were derived from the recorded 2-D patterns by azimuthal integration using the Fit2D v17.006 software^56–58^.

### In situ transmission electron microscopy

A cross-sectional specimen with a final thickness of 50–100 nm was prepared by FIB milling using a Helios Nanolab FIB/SEM (Thermo Fisher Scientific). This specimen was transferred from the ribbon sample to a windowed point on a chip (DENSsolutions). A thin layer of platinum (thickness of 200 nm) was deposited over the target area using an electron beam before the ion-beam exposure. A standard in situ procedure was adopted for lift-out. The sample was secured on the chip with the stage at 45° tilt (in-plane to the chip membrane) with ion-beam-deposited platinum. The chip was then re-oriented with the stage at 10–16° for thinning of the thin foil. Further thinning was conducted until the platinum appeared transparent in a 5 kV electron-beam image, indicating a thin-foil thickness of ∼150 nm. The final thinning was at a reduced voltage of 2 kV to a final electron transparency at 3 kV electron energy. This procedure minimizes the amount of damaged material and reduces the levels of implanted gallium in the final thin foil.

The image and diffraction acquisition and spectroscopic analysis were conducted using a Tecnai Osiris TEM/STEM (FEI) with field-emission gun operated at 200 keV, equipped with a Super-X windowless EDX detector. In situ study was carried out by using a heating holder (DENSsolutions) under the usual TEM vacuum (<10^−8^ mbar). The resistance of the platinum coil in the chip is monitored in a four-point configuration, and the temperature was calculated using calibration constants provided by the manufacturer. The heating holder allowed for controlling the sample temperature with a temperature accuracy of >95%, a temperature homogeneity of >99.5%, and thermal stability of 0.005 K. The details of the calibration are given in ref. ^35^ The temperature ramp had a duration of 3 s for heating and 0.6 s for cooling. Time-series data of SAED diffractions were recorded with an acquisition time of 0.1 s. SAED patterns and associated images were acquired with a Gatan US1000 charge-coupled device camera. The SAED aperture was ~200 nm, and the camera length was set to cover the high-*Q* range up to 12.2 Å^−1^. Due to the absence of thermal drift, data acquisition could be performed during the high-speed heating and cooling process. The chemical composition was analyzed using the EDX detector after crystallization at room temperature.

### Atom probe tomography

Specimens for APT analysis were prepared on a FEI Helios Nanolab 600 focused ion beam milling instrument equipped with a Ga source. APT experiments were carried out on CAMECA LEAP 5000 XR and XS instruments. The atom probe was operated in a laser pulse mode using a repetition rate of 125 kHz, pulse energy of 40 pJ, and samples at a temperature of 60 K. Subsequent APT data reconstruction and post-processing were done using the commercial IVAS 3.8.4 software. Regions containing high and low compositions of different elements were identified by reconstruction via an iso-concentration surface.

### Kikuchi mapping

Transmission Kikuchi diffraction analysis (TKD) was conducted using a Bruker Optimus TKD detector in a Zeiss Merlin field-emission SEM. This system can provide orientation mapping along with phase detection using pre-defined crystal structure inputs of possible phases with a lateral spatial resolution in the order of 10 nm. TKD data were acquired at 30 kV and with 0° sample tilt. Post-processing of TKD data was conducted using the Bruker-ESPIRIT software.

### Auger spectroscopy and X-ray photoelectron spectroscopy

A JAMP 9500 spectrometer (JEOL, Japan) was used for the AES measurements. Electron beam parameters were 10 keV and 10 nA; a spherical electron analyzer was operated in a FRR mode at 0.32%. Concentration quantification was done by taking the peak-to-peak height ratios from differentiated spectra using standard single-element sensitivity factors. An argon-ion beam operated at 1 keV was used for depth profiling; the sputtering rate equivalent was 3.3 nm min^−1^ for SiO_2_.

The XPS measurements were carried out at a PHI 5600 CI system (Physical Electronics, USA). Monochromatized Al *K*-alpha X-rays (350 W) were used and spectra were collected with a hemispherical analyzer operated at 29 eV pass energy. Cleaning by sputtering was done using Ar^+^ ions at 3.5 keV.

## Supplementary information

Supplementary Information

## Data Availability

Source data of the temporal evolution of the XRD patterns presented in Figs. 1, 2, 3, and 4 are available online at (https://archive.materialscloud.org/record/2020.160): 10.24435/materialscloud:nn-38 [ref. ^61^]. All requests for additional data, for example, raw unprocessed data, background, and geometry calibration files, etc., should be sent to Dr Jiri Orava (j.orava@ifw-dresden.de; jiri.orava@ujep.cz) and Dr Ivan Kaban (i.kaban@ifw-dresden.de).

## References

[1] Chen M (2011). A brief overview of bulk metallic glasses. NPG Asia Mater..

[2] Sun Y, Concustell A, Greer AL (2016). Thermomechanical processing of metallic glasses: extending the range of the glassy state. Nat. Rev. Mater..

[3] Qiao JC (2019). Structural heterogeneities and mechanical behavior of amorphous alloys. Prog. Mater. Sci..

[4] Ketov SV (2015). Rejuvenation of metallic glasses by non-affine thermal strain. Nature.

[5] Ross P (2017). Linking macroscopic rejuvenation to nano-elastic fluctuations in a metallic glass. Acta Mater..

[6] Pan J, Ivanov YuP, Zhou WH, Greer AL (2020). Strain-hardening and suppression of shear-banding in rejuvenated bulk metallic glasses. Nature.

[7] Qiao J, Jia H, Liaw PK (2016). Metallic glass matrix composites. Mater. Sci. Eng. R..

[8] Orava J (2019). Fast-heating-induced formation of metallic-glass/crystal composites with enhanced plasticity. Thermochim. Acta.

[9] Wang Y, Chen M, Zhou F, Ma E (2002). High tensile ductility in a nanostructured metal. Nature.

[10] Pauly S, Gorantla S, Wang G, Kühn U, Eckert J (2010). Transformation-mediated ductility in CuZr-based bulk metallic glasses. Nat. Mater..

[11] Villapun VM, Esat F, Bull S, Dover LG, Gonzalez S (2017). Tuning the mechanical and antimicrobial performance of a Cu-based metallic glass composite through cooling rate control and annealing. Materials.

[12] Cullinan T (2015). Mechanisms of isothermal devitrification in amorphous Cu_50_Zr_50_. Metall. Mater. Trans. A.

[13] Kalay I, Kramer MJ, Napolitano RE (2011). High-accuracy X-ray diffraction analysis of phase evolution sequence during devitrification of Cu_50_Zr_50_ metallic glass. Metall. Mater. Trans. A.

[14] Kalay I, Kramer MJ, Napolitano RE (2015). Crystallization kinetics and phase transformation mechanisms in Cu_56_Zr_44_ glassy alloy. Metall. Mater. Trans. A.

[15] Okulov IV (2015). Flash Joule heating for ductilization of metallic glasses. Nat. Commun..

[16] Kosiba K (2017). Transient nucleation and microstructural design in flash-annealed bulk metallic glasses. Acta Mater..

[17] Hajlaoui K (2006). Shear localization and crack blunting of a metallic glass containing nanoparticles: in situ deformation in TEM analysis. Scr. Mater..

[18] Hajlaoui K, Alsaleh N, Alrasheedi NH, Yavari AR (2017). Coalescence and subsequent twinning of nanocrystals during deformation of CuZr-based metallic glasses: the grain size effect. J. Non-Cryst. Solids.

[19] Küchemann S, Samwer S (2016). Ultrafast heating of metallic glasses reveals disordering of the amorphous structure. Acta Mater..

[20] Küchemann S (2016). From ultrafast to slow: heating rate dependence of the glass transition temperature in metallic systems. Philos. Mag. Lett..

[21] Küchemann S (2018). Energy storage in metallic glasses via flash annealing. Adv. Funct. Mater..

[22] Johnson WL (2011). Beating crystallization in glass-forming metals by millisecond heating and processing. Science.

[23] Okulov I (2020). Fabrication of metastable crystalline nanocomposites by flash annealing of Cu_47.5_Zr_47.5_Al_5_ metallic glass using Joule heating. Nanomaterials.

[24] Talaat A, Greve DW, Suraj MV, Ohodnicki PR (2021). Electromagnetic assisted thermal processing of amorphous and nanocrystalline soft magnetic alloys: Fundamentals and advances. J. Alloy. Compd..

[25] Lee D (2016). Crystallization behavior upon heating and cooling in Cu_50_Zr_50_ metallic glass thin films. Acta Mater..

[26] Miao Y, Villarreal R, Talapatra A, Arróyave R, Vlassak JJ (2020). Nanocalorimetry and ab initio study of ternary elements in CuZr-based shape memory alloy. Acta Mater..

[27] Miao Y, Vlassak JJ (2020). Explosive martensitic transformation of supercooled austenite in CuZr-based thin-film shape memory alloys. Acta Mater..

[28] Cheng Q (2020). Phase transformations in a Cu-Zr-Al metallic glass. Scr. Mater..

[29] Silveyra JM, Ferrara E, Huber DL, Monson TC (2018). Soft magnetic materials for a sustainable and electrified world. Science.

[30] Orava J (2020). Fast-current-heating devices to study in situ phase formation in metallic glasses by using high-energy synchrotron radiation. Rev. Sci. Instrum..

[31] Gustmann T (2017). Properties of Cu-based shape-memory alloys prepared by selective laser melting. Shap. Mem. Superelasticity.

[32] Kosiba, K. Flash-annealing of Cu-Zr-Al-based bulk metallic glasses (Ph.D. Thesis, Technische Universität Dresden, Dresden, Germany, 2017).

[33] Wang D (2004). Bulk metallic glass formation in the binary Cu-Zr system. Appl. Phys. Lett..

[34] Herlach DM, Cochrane RF, Egry I, Fecht HJ, Greer AL (1993). Containerless processing in the study of metallic melts and their solidification. Int. Mater. Rev..

[35] Ivanov YuP, Meylan CM, Panagiotopoulos NT, Georgarakis K, Greer AL (2020). In-situ TEM study of the crystallization sequence in a gold-based metallic glass. Acta Mater..

[36] Duan Y (2019). Crystallization behavior of a confined CuZr metallic liquid film with a sandwich-like structure. Phys. Chem. Chem. Phys..

[37] Sohn S, Xie Y, Jung Y, Schroers J, Cha J (2017). Tailoring crystallization phases in metallic glass nanorods via nucleus starvation. Nat. Commun..

[38] Kissinger HE (1957). Reaction kinetics in differential thermal analysis. Anal. Chem..

[39] Orava J, Greer AL (2015). Kissinger method applied to the crystallization of glass-forming liquids: regimes revealed by ultra-fast-heating calorimetry. Thermochim. Acta.

[40] Kokotin V, Hermann H, Eckert J (2011). Computer simulation of the matrix-inclusion interphase in bulk metallic glass based nanocomposites. J. Phys. Condens. Matter.

[41] Orava J, Greer AL (2014). Fast and slow crystal growth kinetics in glass-forming melts. J. Phys. Chem..

[42] Wang Q (2011). Diffusion-controlled crystal growth in deeply undercooled Zr_50_Cu_50_ melt on approaching the glass transition. Phys. Rev. B.

[43] Müller R, Zanotto ED, Fokin VM (2000). Surface crystallization of silicate glasses: nucleation sites and kinetics. J. Non-Cryst. Solids.

[44] Lim KR (2012). Oxidation resistance of the supercooled liquid in Cu_50_Zr_50_ and Cu_46_Zr_46_Al_8_ metallic glasses. J. Mater. Res..

[45] de Campos Neto ND (2019). Phase formation maps in Zr_48_Cu_46.5_Al_4_Nb_1.5_ bulk metallic glass composites as a function of cooling rate and oxygen concentration. Mater. Charact..

[46] Zhou SH, Napolitano RE (2008). Identification of the *B*33 martensite phase in Cu-Zr using first-principles and X-ray diffraction. Scr. Mater..

[47] Zheng J (2018). Phase transformations in equiatomic CuZr shape memory thin films analyzed by differential nanocalorimetry. Acta Mater..

[48] Sahu KK (2010). Phase separation mediate devitrification of Al_88_Y_7_Fe_5_ glasses. Acta Mater..

[49] Wu Y (2011). Formation of Cu-Zr-Al bulk metallic glass composites with improved tensile properties. Acta Mater..

[50] Kargl F, Weis H, Unruh T, Meyer A (2012). Self diffusion in liquid aluminium. J. Phys. Conf. Ser..

[51] Balachandran S (2019). Elemental re-distribution inside shear bands revealed by correlative atom-probe tomography and electron microscopy in a deformed metallic glass. Scr. Mater..

[52] Boucharat N, Hebert R, Rösner H, Valiev R, Wilde G (2005). Nanocrystallization of amorphous Al_88_Y_7_Fe_5_ alloy induced by plastic deformation. Scr. Mater..

[53] Chellali MR, Nandam SH, Hahn H (2020). Deformation-induced chemical inhomogeneity and short-circuit diffusion in shear bands of a bulk metallic glass. Phys. Rev. Lett..

[54] Kaban I (2015). Atomic structure and formation of CuZrAl bulk metallic glasses and composites. Acta Mater..

[55] Guo W, Saida J, Zhao M, Lü S, Wu S (2019). Non-thermal crystallization process in heterogeneous metallic glass upon deep cryogenic cycling treatment. J. Mater. Sci..

[56] Hammersley AP (1995). Calibration and correction of distortions in two-dimensional detector systems. Rev. Sci. Instrum..

[57] Hammersley AP, Svensson SO, Hanfland M, Fitch AN, Häusermann D (1996). Two-dimensional detector software: from real detector to idealised image or two-theta scan. High. Press. Res..

[58] Hammersley, A. P. ESRF Internal Report, ESRF98HA01T, FIT2D V9.129 Reference Manual V3.1, (1998).

[59] Degen T, Sadki M, Bron E, König U, Nénert G (2014). The HighScore suite. Powder Diffr..

[60] Shuleshova O (2018). Metastable phase formation in undercooled Fe-Co melts under terrestrial and microgravity conditions. IFW Dresd.: Annu. Rep..

[61] Orava J (2020). In situ high-energy X-ray diffraction of a CuZr-based metallic glass. Mater. Cloud Arch..

