# Supplementary material for: In situ correlation between metastable phase-transformation mechanism and kinetics in a metallic glass
Source: Nat Commun. 2021 May 14;12:2839. doi: 10.1038/s41467-021-23028-9 (PMC8121901; doi:10.1038/s41467-021-23028-9)
Supplement: Supplementary file 1 — Supplementary Information [file 41467_2021_23028_MOESM1_ESM.pdf]

In situ correlation between metastable phase-transformation mechanism and kinetics in a metallic glass

Jiri Orava,[1,†,*] Shanoob Balachandran,[2,*] Xiaoliang Han,[1,*] Olga Shuleshova,[1] Ebrahim Nurouzi,[2] Ivan Soldatov,[3,4] Steffen Oswald,[1] Olof Gutowski,[5] Oleh Ivashko,[5] Ann-Christin Dippel,[5] Martin v. Zimmermann,[5] Yurii P. Ivanov,[6] A. Lindsay Greer,[6] Dierk Raabe,[2] Michael Herbig[2] and Ivan Kaban[1,*]

[1] *IFW Dresden, Institute for Complex Materials, Helmholtzstr. 20, Dresden 010 69, Germany*
[2] *Max-Planck-Institut für Eisenforschung, Max-Planck-Str. 1, Düsseldorf 402 37, Germany*
[3] *IFW Dresden, Institute for Metallic Materials, Helmholtzstr. 20, Dresden 010 69, Germany*
[4] *Institute of Natural Sciences, Ural Federal University, 620002 Ekaterinburg, Russia*
[5] *Deutsches Elektronen-Synchrotron DESY, Notkestr. 85, Hamburg 226 03, Germany*
[6] *Department of Materials Science and Metallurgy, University of Cambridge, Charles Babbage Rd. 27, Cambridge CB3 0FS, United Kingdom*

[†] present address: *Faculty of Environment, Jan Evangelista Purkyne University in Usti nad Labem, Pasteurova 3632/15, Usti nad Labem 400 96, Czech Republic*

Corresponding authors email: jiri.orava@ujep.cz (JO) at Jan Evangelista Purkyne University in Usti nad Labem; s.balachandran@mpie.de (SB) at MPIE Düsseldorf; x.han@ifw-dresden.de (XH) and i.kaban@ifw-dresden.de (IK) at IFW Dresden

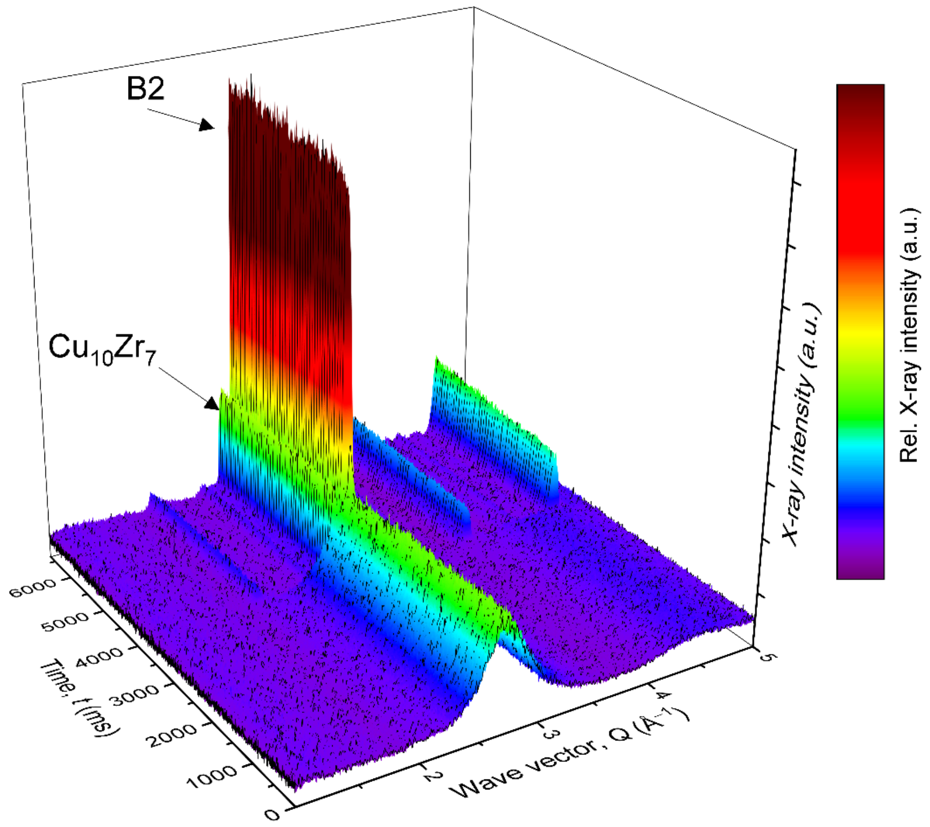

Supplementary Figure 1. 3-D representation of the phase transformations for crystallization of $Cu_{47.5}Zr_{47.5}Al_{5.0}$ ribbons at a subcritical heating rate of $\Phi \approx 100–150$ K s^{-1}. Only the most intense peak for each phase is labelled.

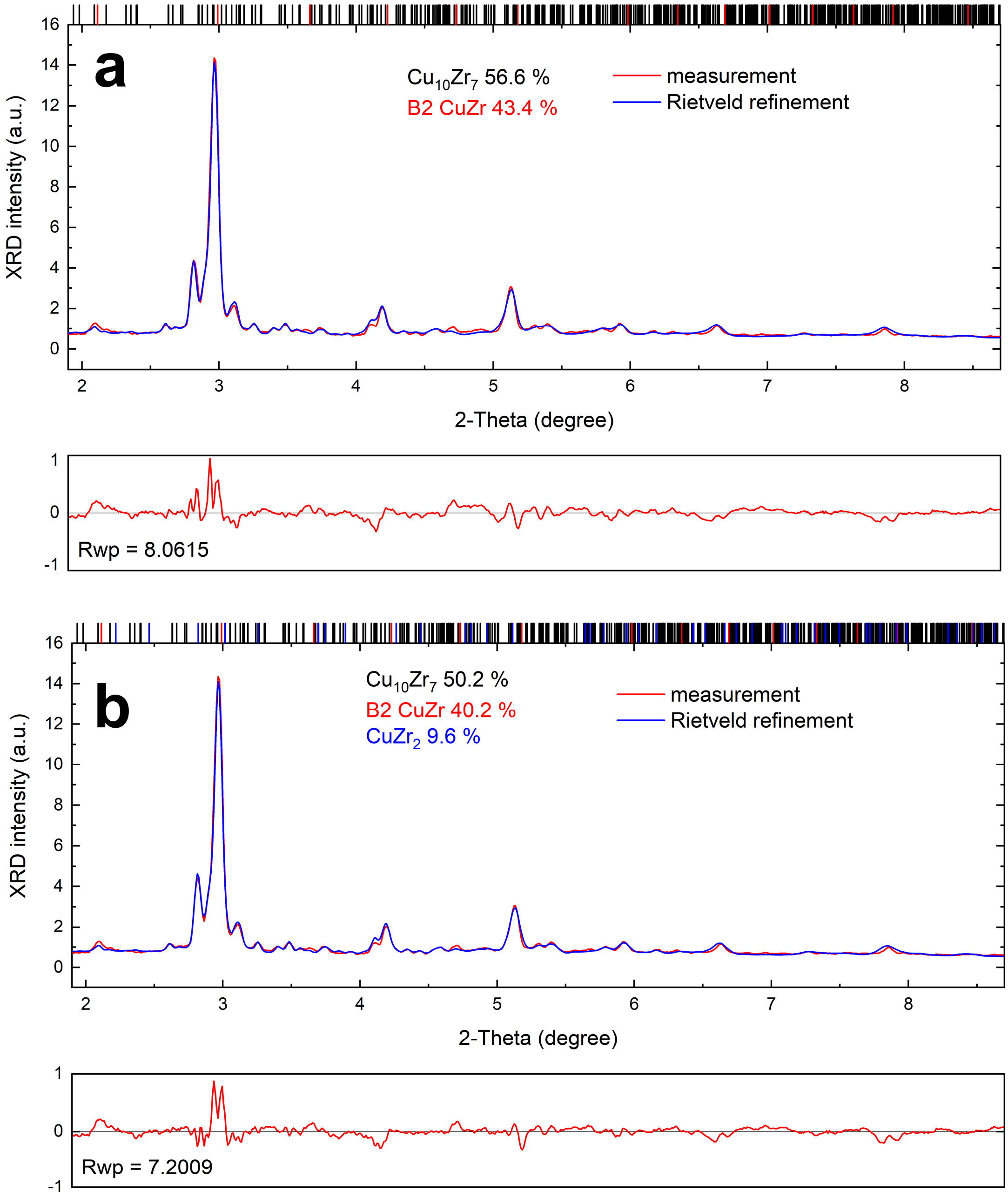

Supplementary Figure 2. Rietveld refinement of 20 summed high-energy XRD patterns taken around point 4 in Figure 1a of the subcritical heating. The patterns correspond to t = 3268–3704 ms and $T \approx$ 1007–1013 K. **a** A best-fit of the two dominant phases, i.e., $Cu_{10}Zr_7$ and B2. **b** Adding the $CuZr_2$ phase to the analysis may hint at its possible presence as shown around a 2-Theta range of 2.5–3.2 deg (corresponding to a Q-range of 2.7–3.2 Å in Figure 1). Nevertheless, while the overall fit quality is improved, the fitting unambiguity does not permit evidencing the phase directly.

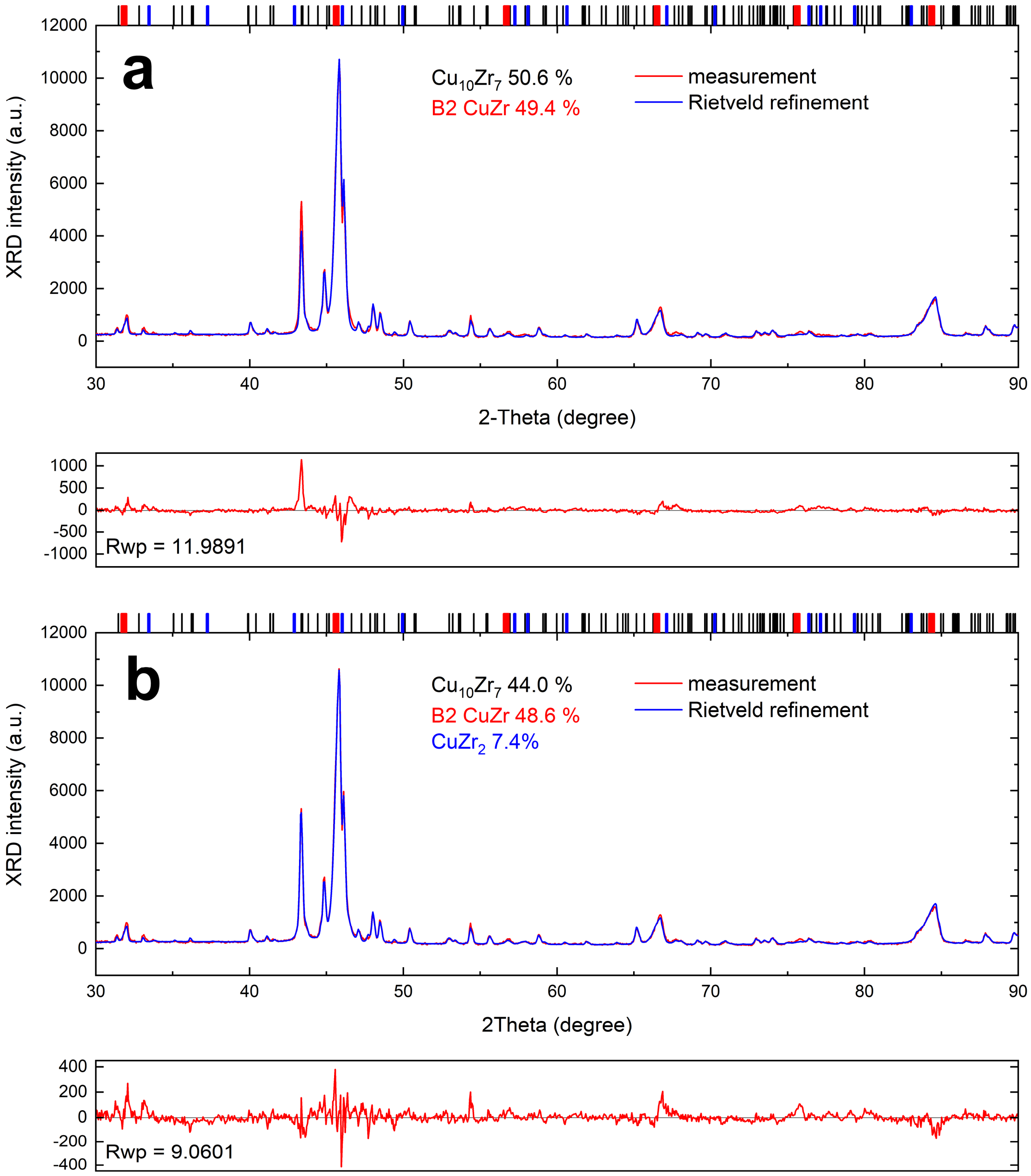

Supplementary Figure 3. Rietveld refinement of room-temperature laboratory XRD intensity of the subcritical-heating microstructure obtained via heating/cooling conditions given in Figure 1. a Rietveld refinement by using the two main phases $Cu_{10}Zr_7$ and B2. **b** Similar to Supplementary Figure 2, an improved fit quality can be achieved by considering $CuZr_2$ phase, which gives a hint at the possible presence of the phase in the final microstructure, yet, its content must be rather low. The laboratory XRD measurements was by using a X'Pert Pro DY2183 XRD equipped with a Co X-ray tube.

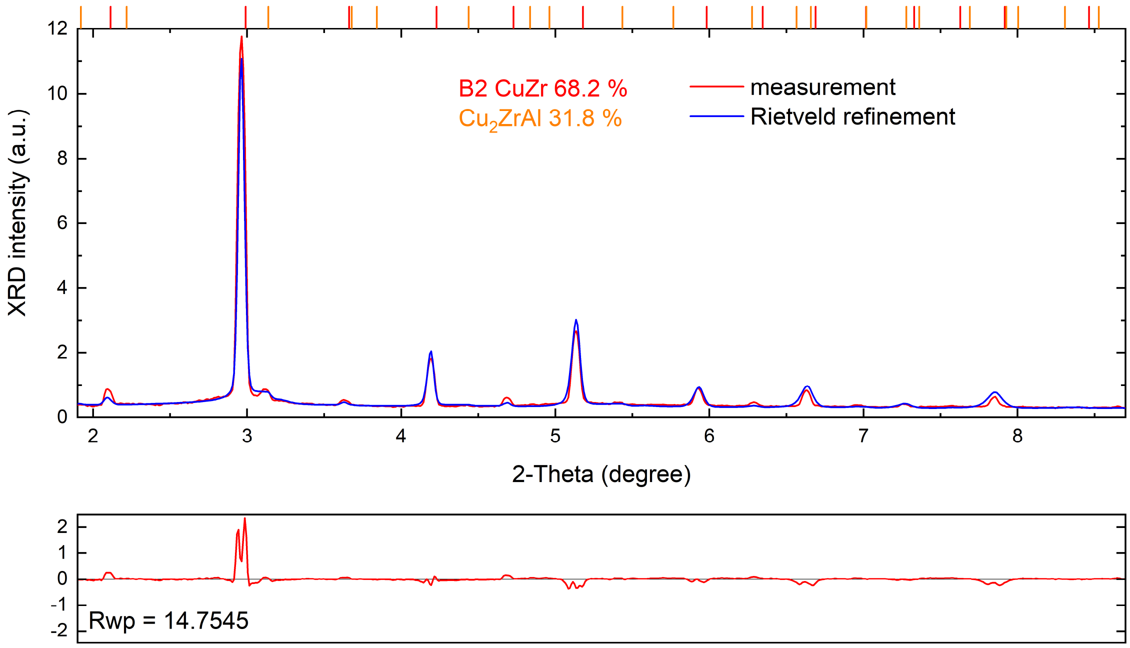

Supplementary Figure 4. Rietveld refinement of 10 summed high-energy XRD patterns taken around point 4 in Figure 2a of the supercritical heating. Only two high-temperature B2 and Cu_2ZrAl phases were identified. The summed intensities correspond to t = 552–589 ms and $T \approx$ 1103–1145 K of the heating step presented in Figure 2a.

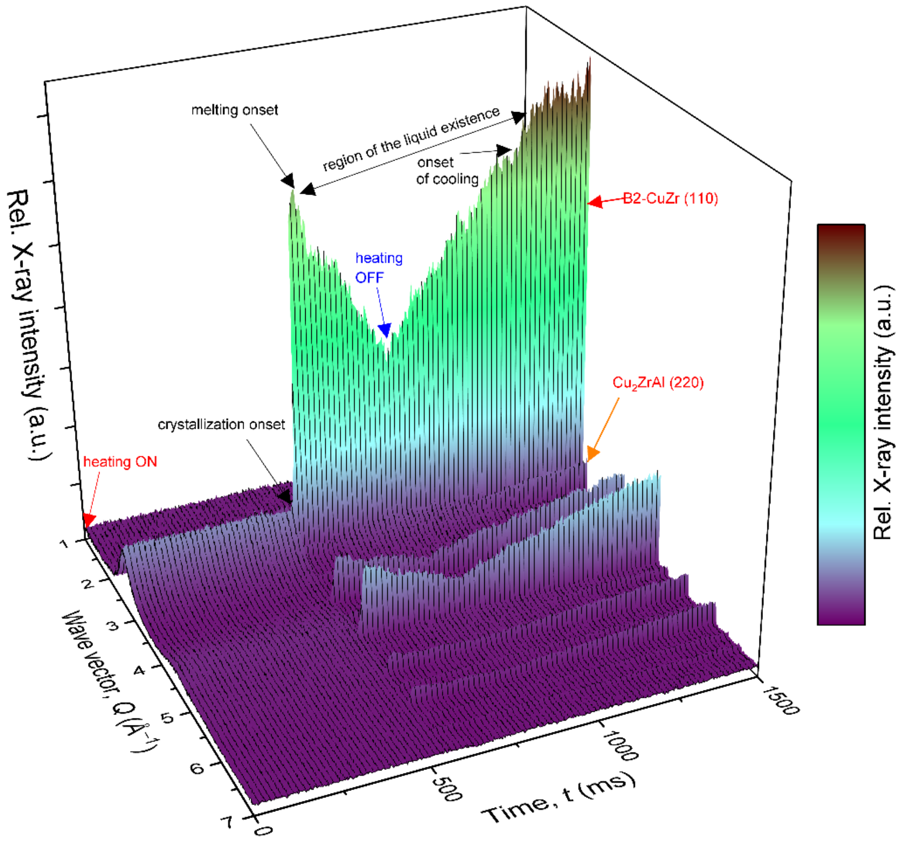

Supplementary Figure 5. 3-D representation of the phase transformations for crystallization, partial melting and solidification regions before the onset of Newtonian cooling presented in Figure 2 (the main manuscript). Thermal events are marked with the arrows and labeled accordingly.

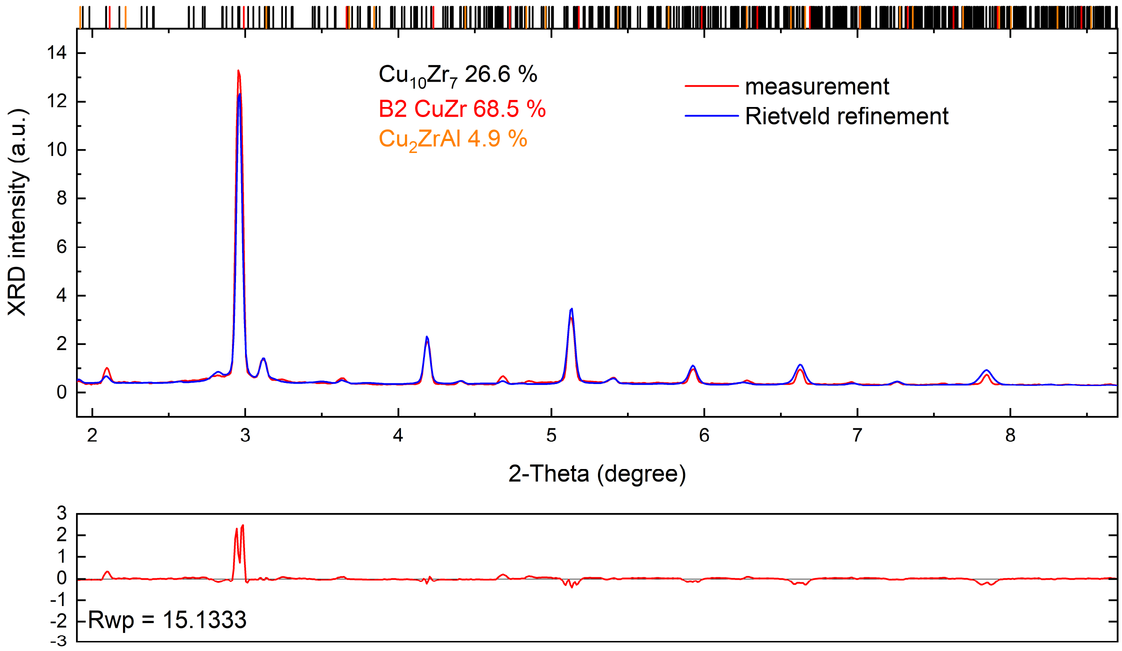

Supplementary Figure 6. Rietveld refinement of 10 summed high-energy XRD patterns taken around point 5 in Figure 2a on cooling. Along with the present high-temperature B2 and Cu_2ZrAl phases, the $Cu_{10}Zr_7$ formation on cooling was resolved. The summed intensities correspond to t = 1300–1336 ms and $T \approx$ 1132–1121 K.

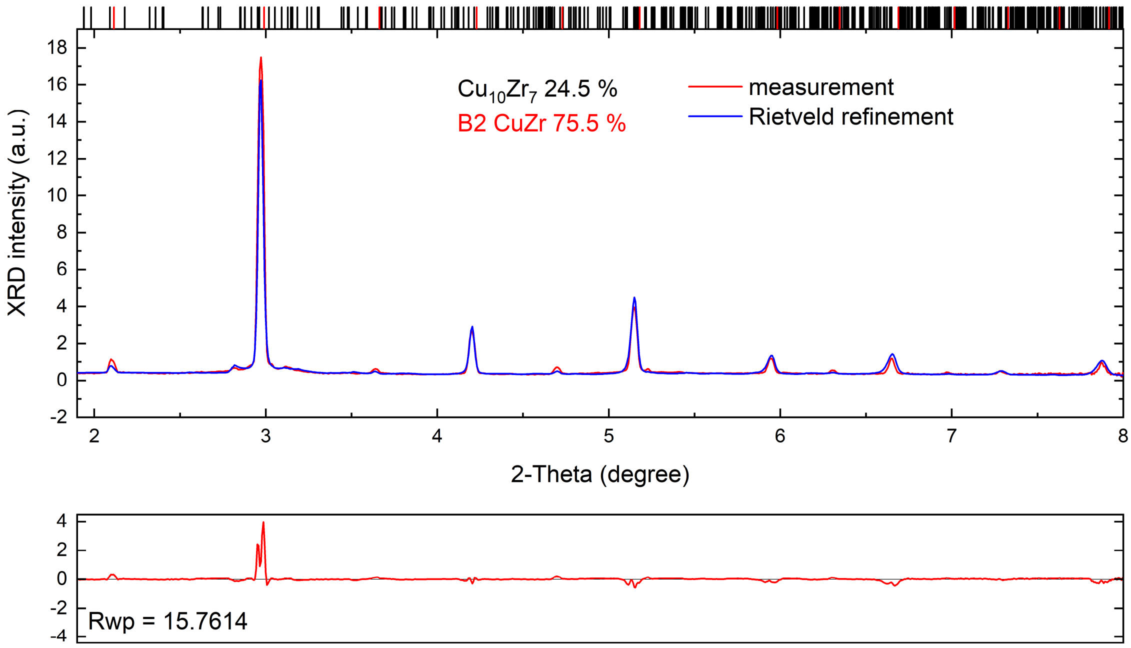

Supplementary Figure 7. Rietveld refinement of 20 summed high-energy XRD patterns taken around point 4 in Figure 3a on heating the glass inside a chamber flushed with He. The B2 CuZr was the predominant phase, and the minor content of $Cu_{10}Zr_7$ phase could be resolved as well. The summed intensities correspond to t = 609–686 ms and $T \approx$ 978–1024 K.

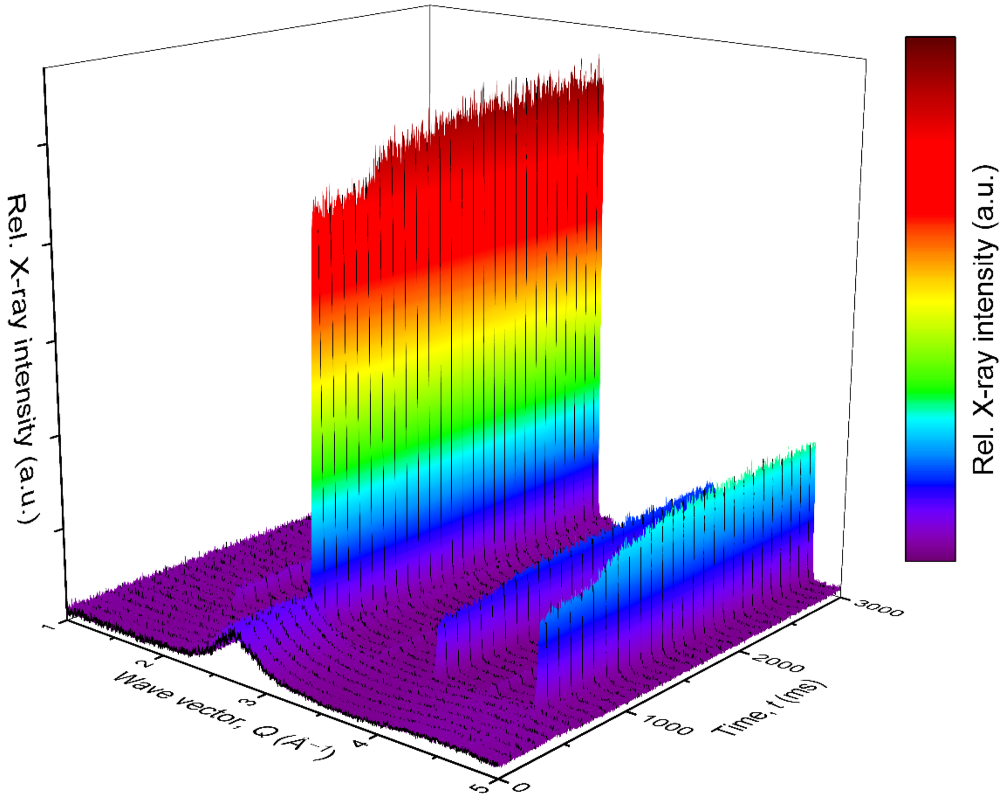

Supplementary Figure 8. A 3-D representation of phase transformations in a chamber flushed with He as presented in Figure 3 of the main text. A heating rate was $\Phi \approx 1000$ K s^{-1} and cooling rate was 1500–1800 K s^{-1}. The most intense peak corresponds to the B2 phase.

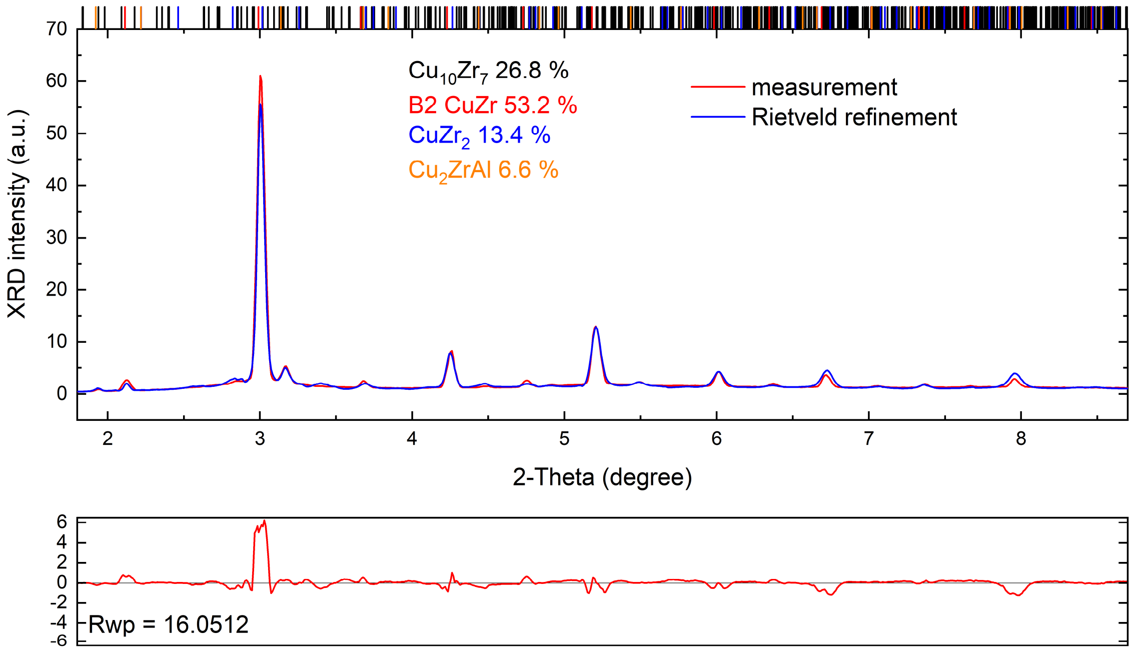

Supplementary Figure 9. Rietveld refinement of the high-energy XRD pattern taken at point 4 shown in Figure 4a, *t* = 50.2 s and *T* = 1134 K, during solidification of supercooled $Cu_{47.5}Zr_{47.5}Al_{5.0}$ liquid in EML facility.

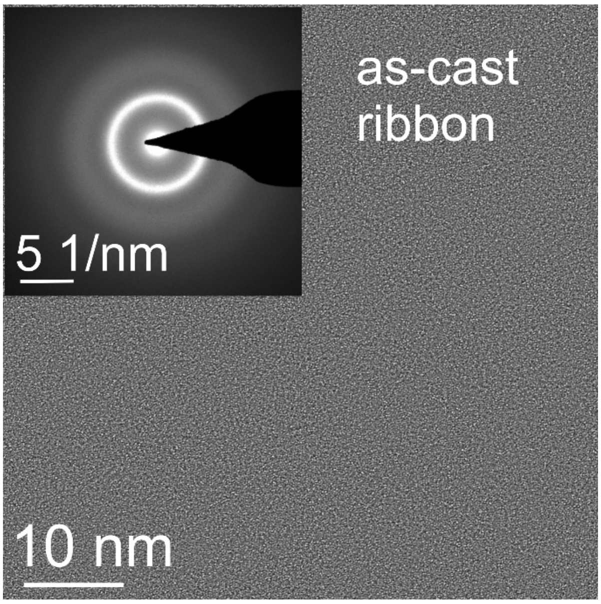

Supplementary Figure 10. High-resolution TEM and the corresponding SAED pattern (inset) of the as-spun $Cu_{47.5}Zr_{47.5}Al_{5.0}$ ribbon.

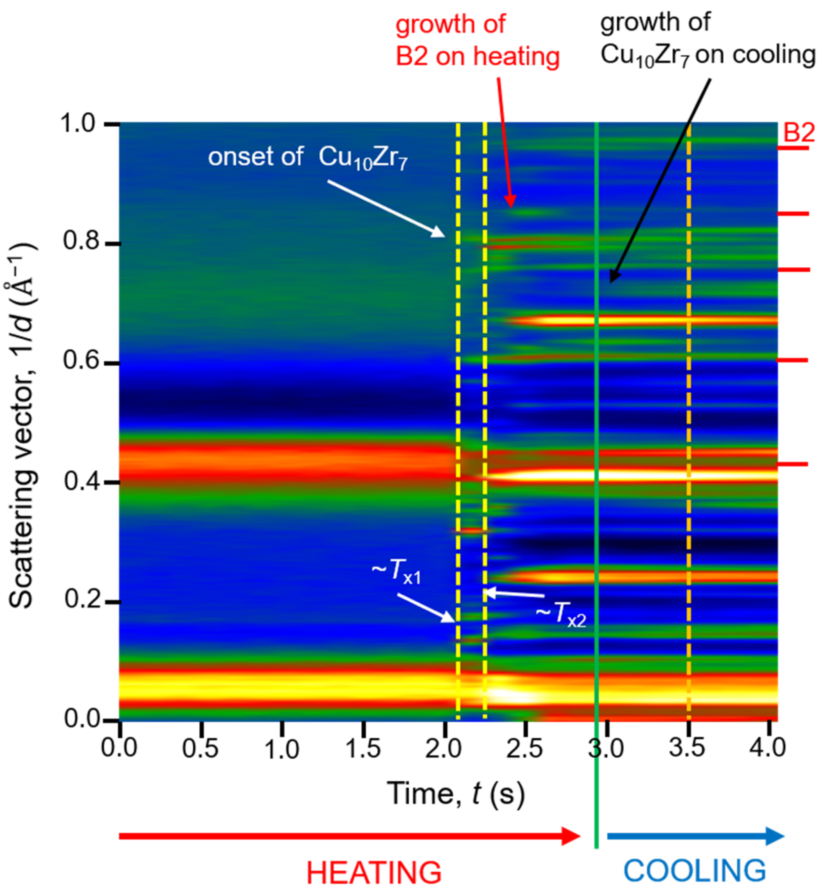

Supplementary Figure 11. Integrated temporal evolution of SAED patterns on heating/cooling from Figure 5 of the main text. The intensity map is aimed to qualitatively show the phase evolution of the crystallization kinetics presented in Figure 5a. No background was subtracted and the texture change during various crystallization steps was not considered.

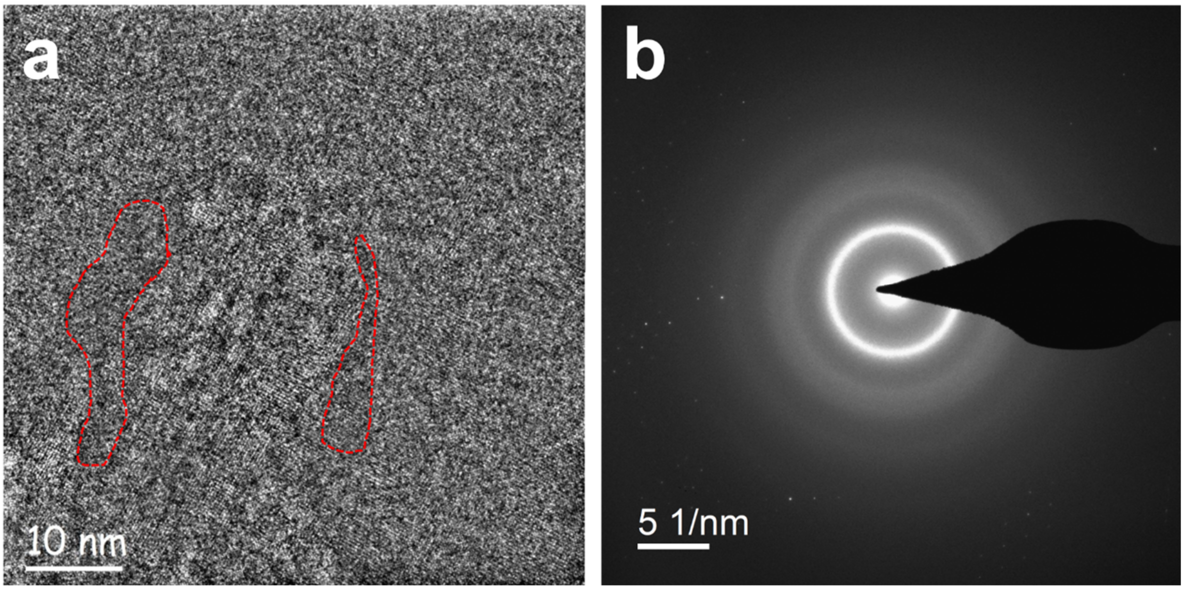

Supplementary Figure 12. Confirmation of the presence of amorphous regions in the final microstructure shown in Figure 5. a. HRTEM image: the largest amorphous regions are marked by the areas enclosed in red. **b.** The corresponding SAED pattern confirming the amorphicity – the spots visible at high *Q* are due to the crystals in the nearby area.

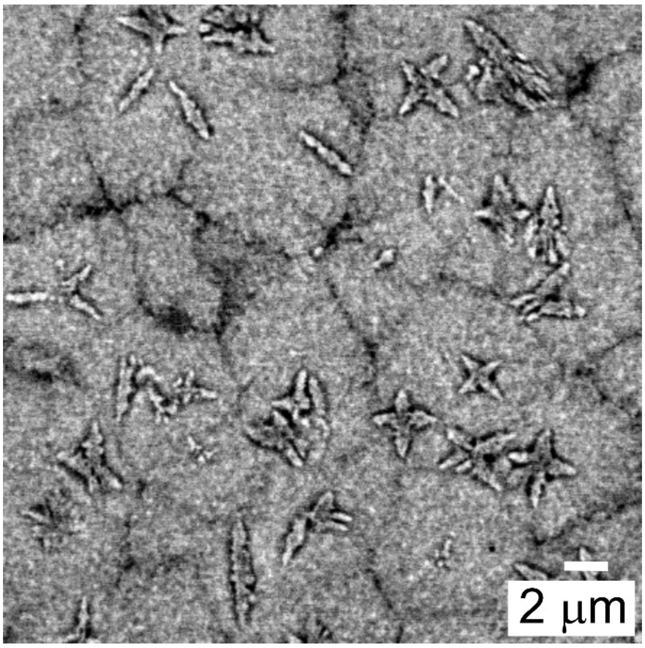

Supplementary Figure 13. A representative SEM micrograph of a fully-crystallized sample prepared in a vacuum. Dendrites of $Cu_{10}Zr_7$ phase are surrounded by spheroidal grains of B2 phase. The scale bar is 2 μm. The specimen was prepared by grinding and polishing with diamond suspensions, particle sizes of 1 and then 0.25 μm, prior to SEM imaging.

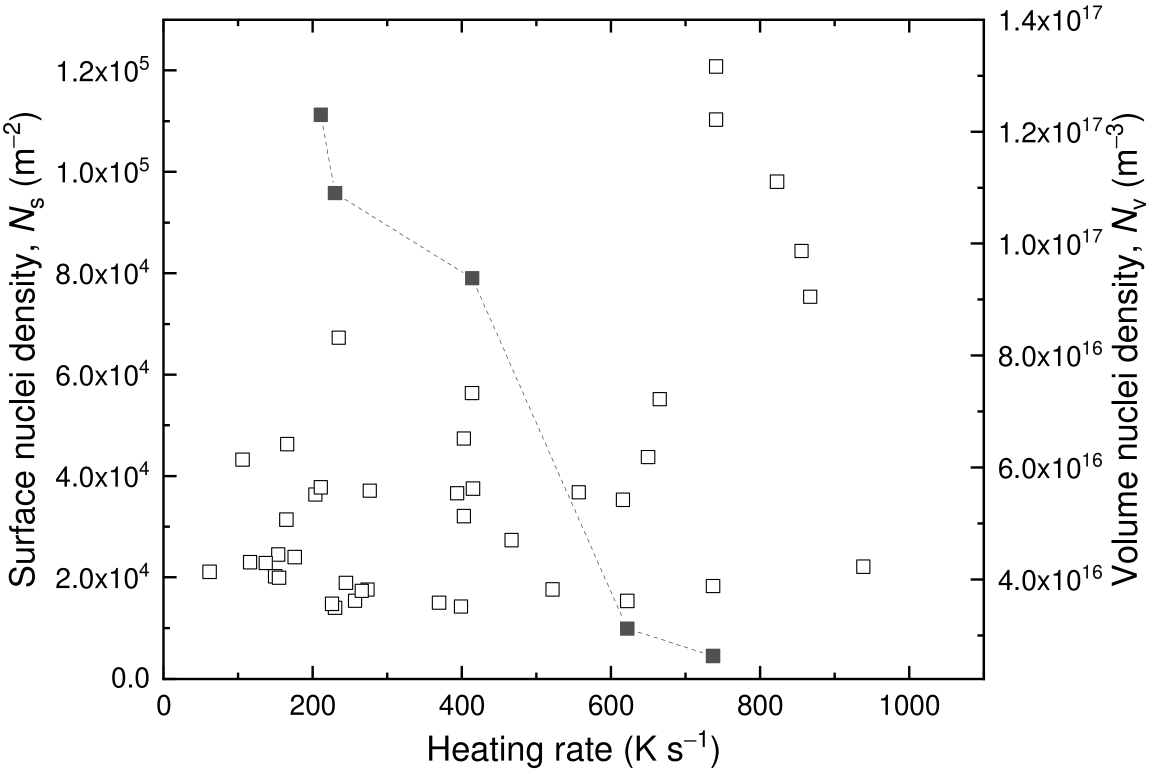

Supplementary Figure 14. Dependence of surface nuclei density N_s (open symbols) and volume nuclei density N_v (full symbols) on Φ during flash-annealing of $Cu_{47.5}Zr_{47.5}Al_{5.0}$ metallic-glass ribbons in a vacuum.

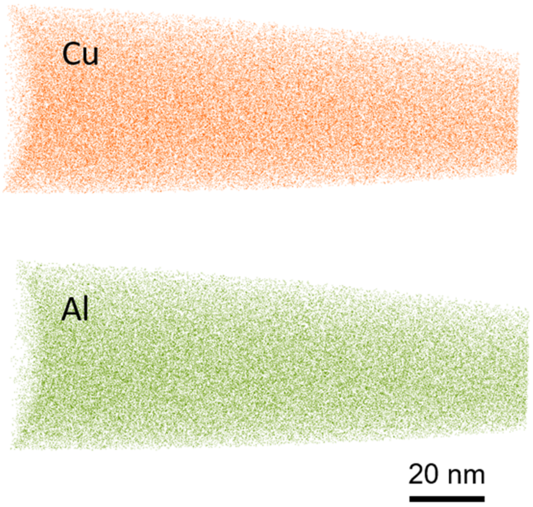

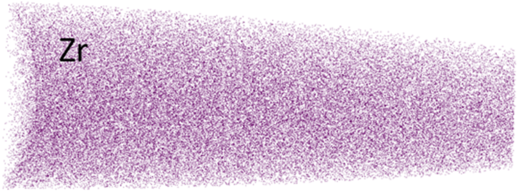

element	at.% (±0.5 at.%)
Cu	46.5
Zr	47.0
Al	5.3

Supplementary Figure 15. Atom probe tomography 3-D atom maps of the as-spun $Cu_{47.5}Zr_{47.5}Al_{5.0}$ glass. Elements are distributed homogeneously. A composition close to the nominal one is confirmed.

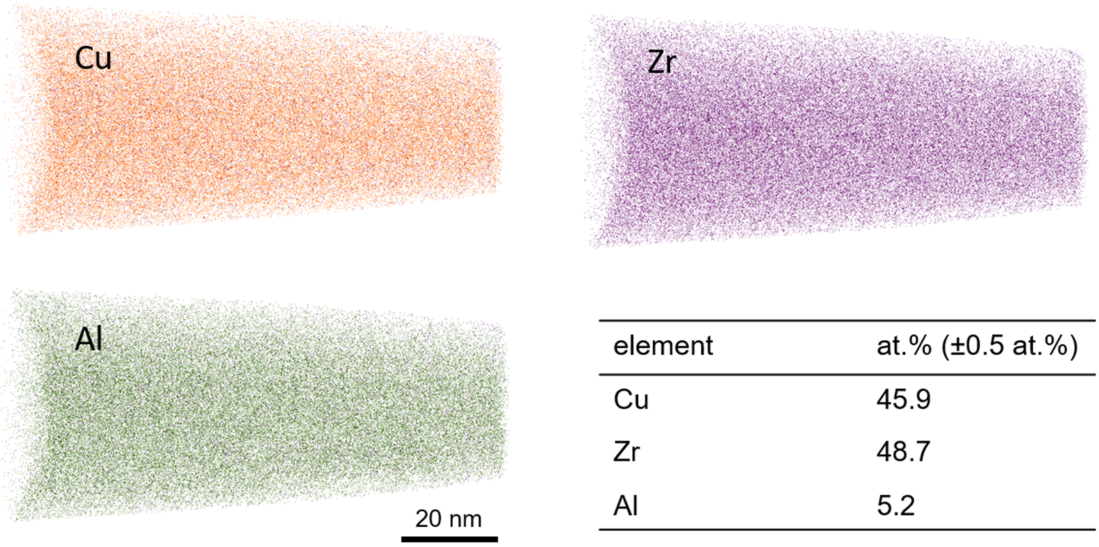

element	at.% (±0.5 at.%)
Cu	45.9
Zr	48.7
Al	5.2

Supplementary Figure 16. Atom probe tomography composition map of a B2 phase. The composition confirms the near CuZr stoichiometry of the crystal.

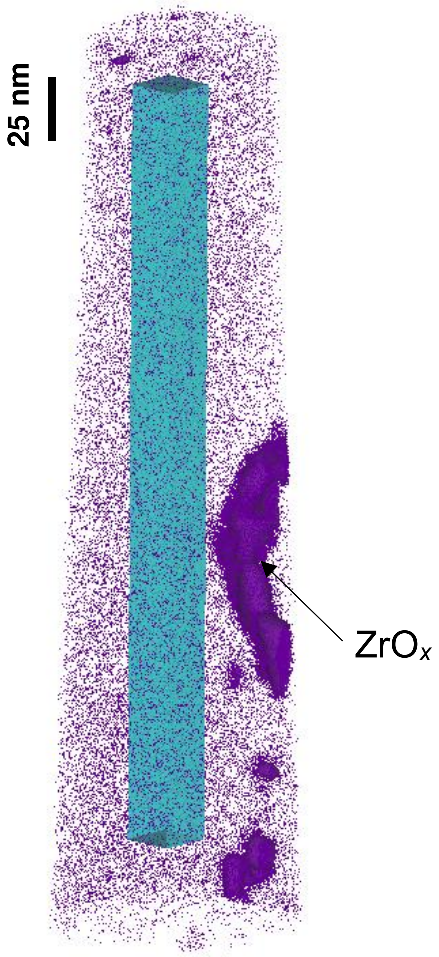

Supplementary Figure 17. An interface between two crystalline phases from Figure 7c is prone to oxidation and contains a ZrO_x oxide layer up to ~20 nm thick.

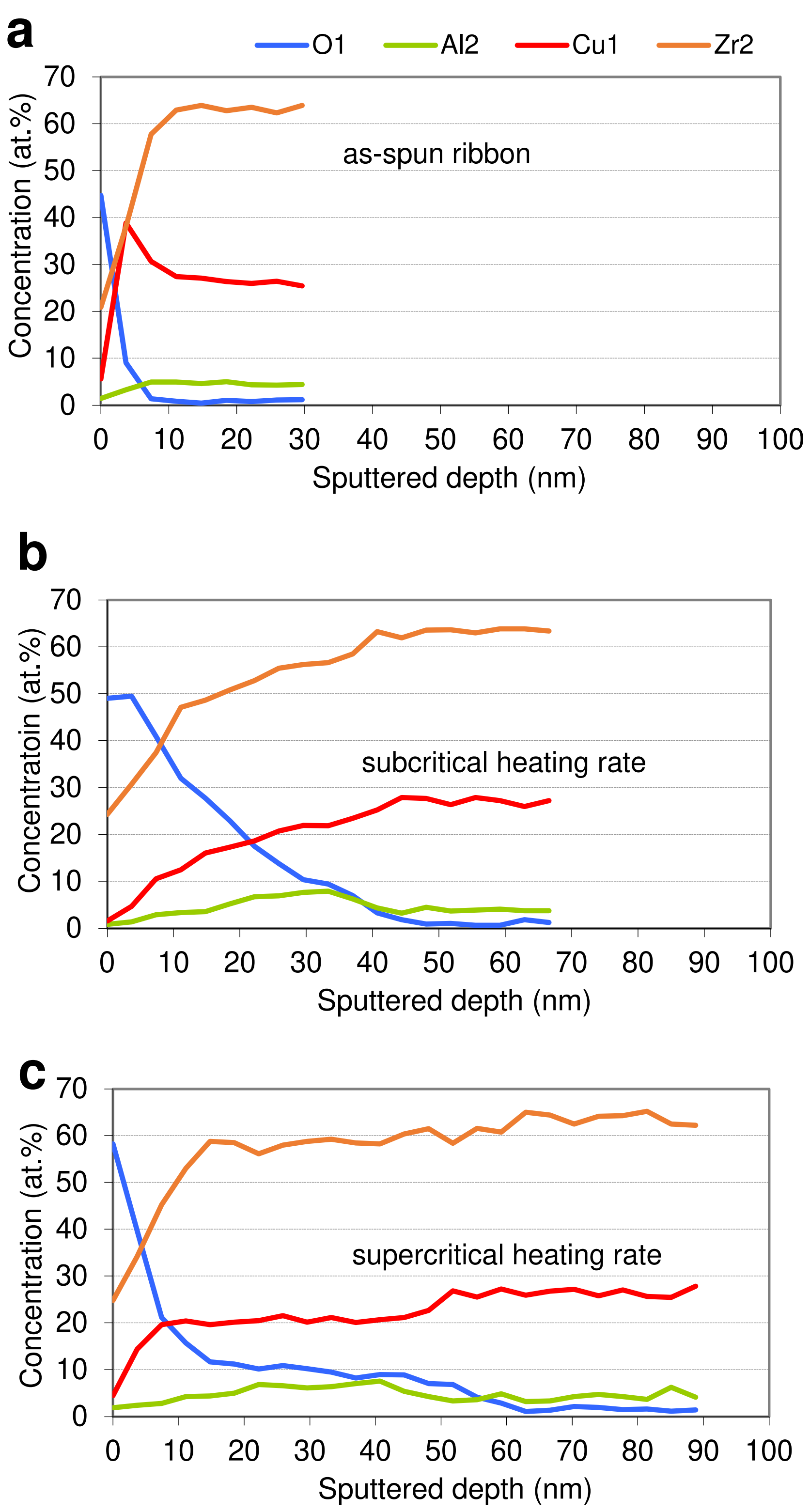

Supplementary Figure 18. Auger spectra from the surface of metallic-glass ribbons: a an as-spun ribbon, and after heating under vacuum at **b** subcritical and **c** supercritical heating rate. The focus is only on qualitative changes, compositional trends, of different elements. The sputtering yields of different elements have not been calibrated; the sensitivity factors are for pure elements and quantitative evaluation, the absolute concentration, is not possible. Trace elements (C, Si…) are not shown.

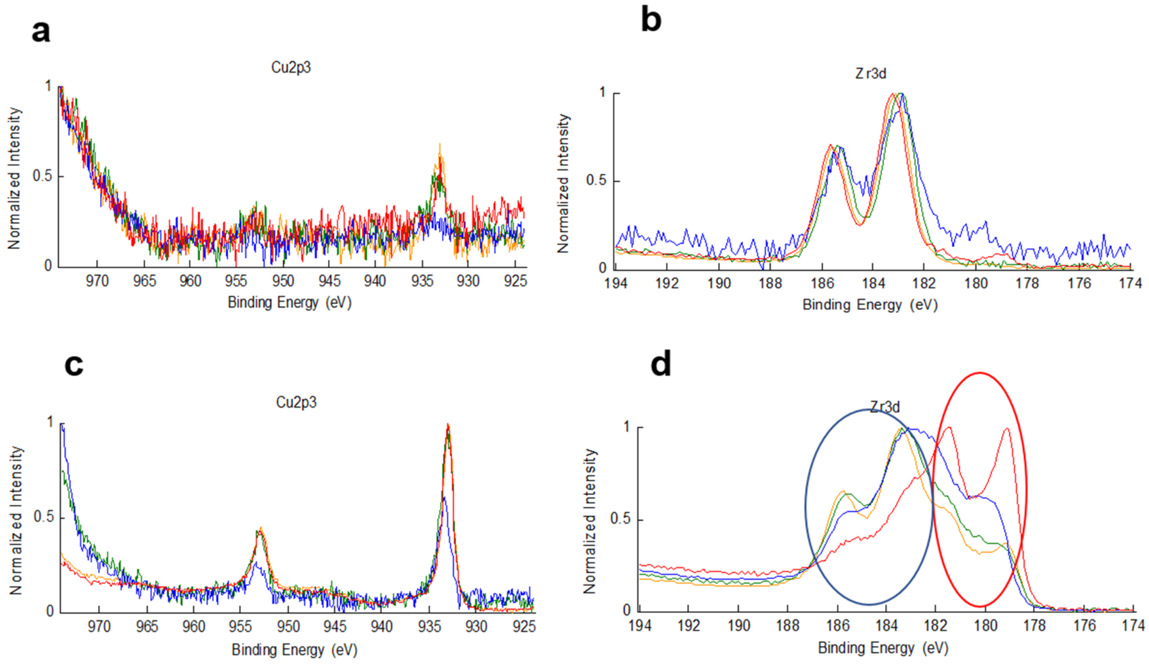

Supplementary Figure 19. Representative XPS spectra highlighting the bonding form of Zr and Cu in a crystalline sample after heating in a vacuum and in a chamber flushed with He. a, **b** pristine surface; **c**, **d** surface after sputtering (blue curve – samples made in a chamber flushed with He; remaining colored curves – samples made in a vacuum). In part **(d)**, the zirconium metallic and oxide states are highlighted by red and blue ellipses, respectively.

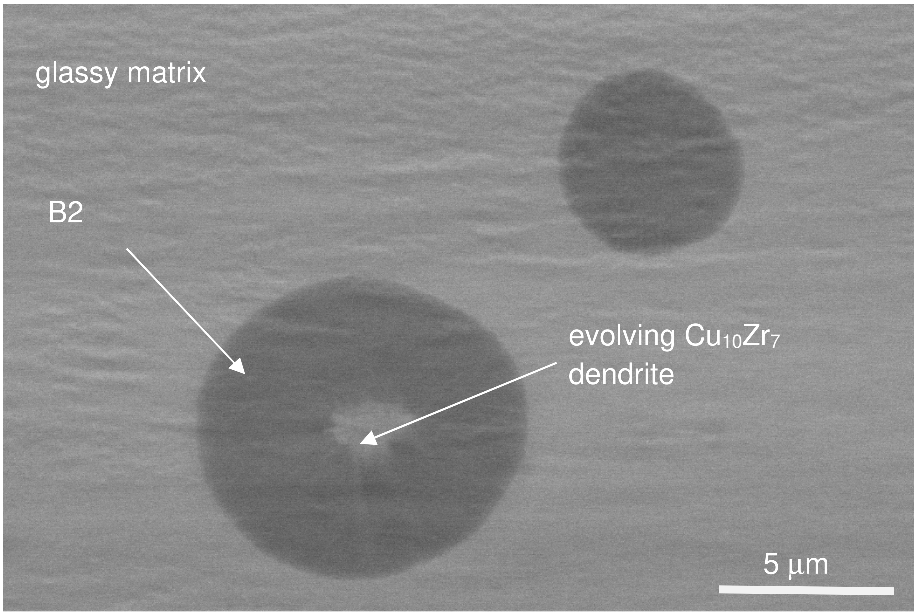

Supplementary Figure 20. A representative SEM micrograph of a pristine surface of a glass-crystal composite made in a chamber flushed with He.

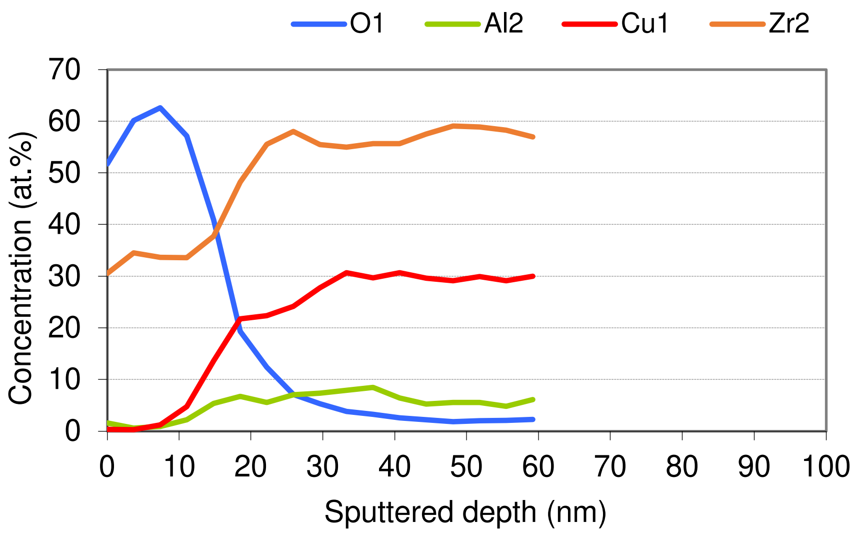

Supplementary Figure 21. Auger spectroscopy of a crystalline ribbon made by heating in a chamber flushed with He. The focus is only on qualitative changes, compositional trends, of different elements. The sputtering yields of different elements have not been calibrated, the sensitive factors are for pure elements and quantitative evaluation, the absolute concentration, is not possible. Trace elements (C, Si…) are not shown.
